# The matrix proteins aggrecan and fibulin-1 play a key role in determining aortic stiffness

**DOI:** 10.1038/s41598-018-25851-5

**Published:** 2018-06-04

**Authors:** Yasmin, Raya Al Maskari, Carmel M. McEniery, Sarah E. Cleary, Ye Li, Keith Siew, Nichola L. Figg, Ashraf W. Khir, John R. Cockcroft, Ian B. Wilkinson, Kevin M. O’Shaughnessy

**Affiliations:** 1 Division of Experimental Medicine & Immunotherapeutics, University of Cambridge, Addenbrooke’s Hospital, Cambridge, UK; 2 Brunel Institute of Bioengineering, Brunel University, Uxbridge, Middlesex UK; 3 Division of Cardiovascular Medicine, University of Cambridge, Addenbrooke’s Hospital, Cambridge, UK; 4 Division of Cardiology, New York-Presbyterian Hospital, Columbia University, New York, USA

## Abstract

Stiffening of the aorta is an important independent risk factor for myocardial infarction and stroke. Yet its genetics is complex and little is known about its molecular drivers. We have identified for the first time, tagSNPs in the genes for extracellular matrix proteins, aggrecan and fibulin-1, that modulate stiffness in young healthy adults. We confirmed SNP associations with *ex vivo* stiffness measurements and expression studies in human donor aortic tissues. Both aggrecan and fibulin-1 were found in the aortic wall, but with marked differences in the distribution and glycosylation of aggrecan reflecting loss of chondroitin-sulphate binding domains. These differences were age-dependent but the striking finding was the acceleration of this process in stiff versus elastic young aortas. These findings suggest that aggrecan and fibulin-1 have critical roles in determining the biomechanics of the aorta and their modification with age could underpin age-related aortic stiffening.

## Introduction

Stiffening of the large arteries such as the aorta (arteriosclerosis) has several important adverse haemodynamic consequences, including widening of the pulse pressure and altered shear stress. These promote vascular and cardiac remodelling, and ultimately cause increased cardiovascular (CV) endpoints. Aortic pulse wave velocity (aPWV) is the gold-standard measure of stiffness. It is also an important independent predictor of clinical CV outcomes^1^, including myocardial infarction, heart failure and stroke^2,3^ which make it an attractive target for therapeutic intervention.

Like most cardiovascular traits, aortic stiffness has a multi-factorial aetiology and a polygenic pattern of inheritance (~40% heritability). This reflects the impact of many genes that influence processes such as cell signalling, the cytoskeleton, mechanical regulation of vascular structure^4^, and vascular smooth muscle tone^5^. Nonetheless, dissecting genetic influences is challenging and the precise molecular players and pathways remain elusive. A GWAS meta-analysis by the AortaGen consortium, identified several genetic variants on chromosome 14 that strongly associate with aortic stiffness^6^, but this locus lies in a gene desert with BCL11B (1 MB telomeric from the locus) as a the most plausible candidate in the region. However, the biological significance of this GWAS signal is still unclear.

**Figure 1:**
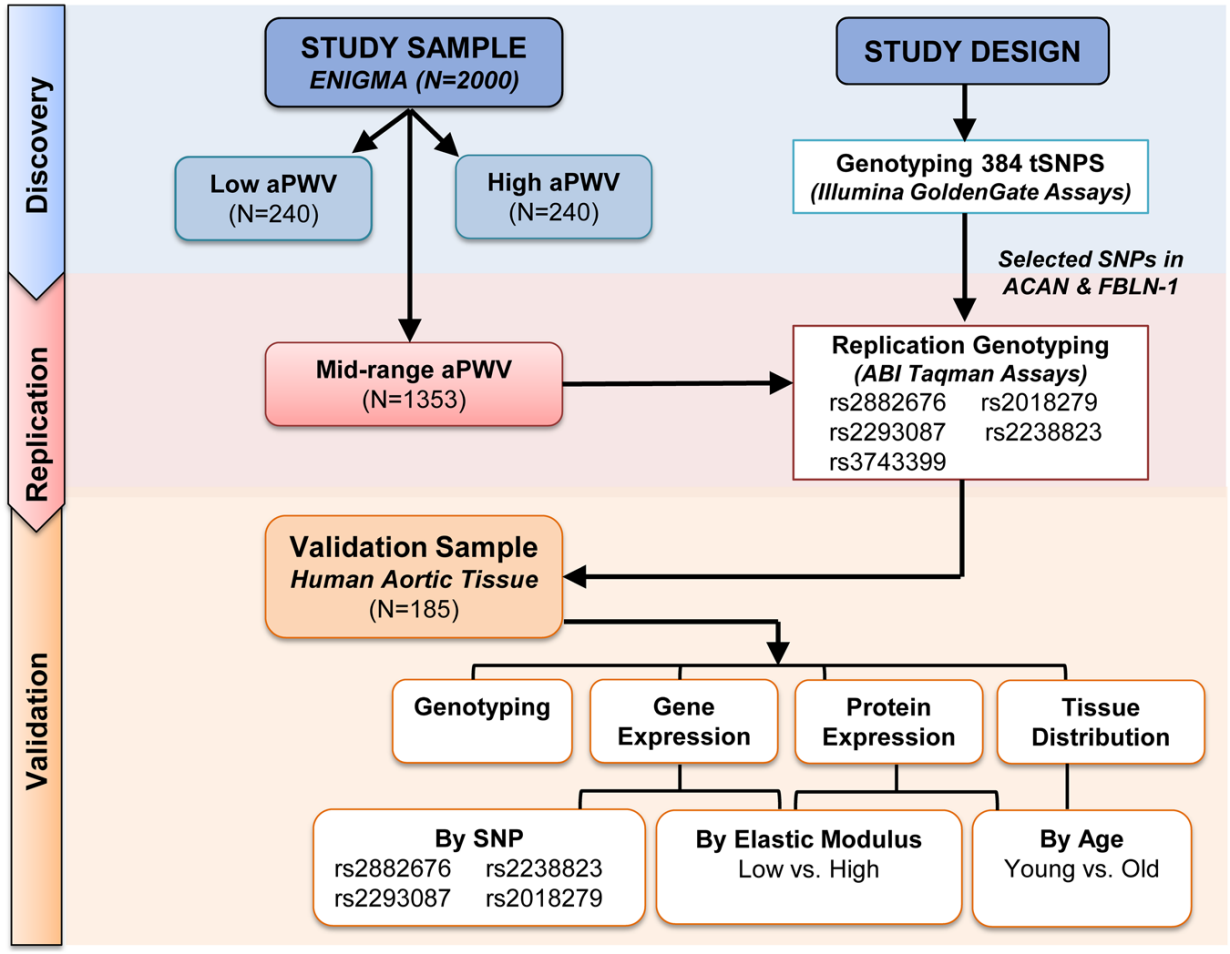
Study Flow. Design and samples investigated.

To date, all the genetic studies on stiffness have focused on older adults (>45 years)^6^, so the observed associations may relate to environmental exposures (e.g. smoking and hypertension) and atherosclerosis. However, with ageing, arteries stiffen as a consequence of a number of processes including elastin degradation, extracellular matrix (ECM) remodelling, calcification of the elastic lamellae, vascular smooth muscle cell senescence and apoptosis, inflammation and oxidative stress^7–9^. So, to identify genetic risk alleles for stiffness it is key to study relatively young people to isolate ‘pure’ genetic contribution and minimise atherosclerotic related influences^10,11^. To this end, we have performed a candidate-gene based association study using the phenotypic extremes from a cohort of young healthy adults (the ENIGMA cohort) with low cardiovascular risk. To do this, we chose subjects with low versus high aPWV values and compared them using tagging single nucleotide polymorphisms (tagSNPs) to efficiently capture the genetic information from the linkage disequilibrium (LD) or haplotype blocks of the genes of interest^12^. We then validated significant associations in the remaining ENIGMA subjects. Finally, we correlated validated SNPs with an *ex vivo* stiffness measurement in human donor aortic tissues and explored the tissue expression of these SNPs and their encoded proteins (the study plan is shown in Fig. 1).

## Results

### SNP Analysis in ENIGMA

We performed genetic association studies using a discovery cohort pre-selected for extremes of aPWV from the ENIGMA study. The discovery subjects represented the top and bottom 12% of the study (n = 240 in each extreme group), and tagSNPs were from genes correlated with arterial wall properties in the gene profiling study by Durier *et al*.^4^ and other published studies^5^ (Supplementary Table 1, see Supplementary Material for references). We found that the two groups in the primary study chosen based on their extreme aPWV were actually well matched for age, gender, body mass index and systolic pressure (Table 1). However, diastolic and mean pressures, heart rate and aPWV were significantly different between the two extreme groups.

**Table 1:** Baseline characteristics of ENIGMA cohort.

Parameters	Study Groups		Significance level (p)
	Top*	Bottom*	
Age (y)	21 ± 3	20 ± 2	ns
Male/Female (n)	120/120	120/120	ns
Non-Smokers (n)	233	232	—
Alcohol-Non drinkers (n)	25	30	—
BMI (kg/m^2^)	23 ± 4	22 ± 3	ns
SBP (mmHg)	118 ± 14	116 ± 13	ns
DBP (mmHg)	69 ± 8	67 ± 8	0.015
MAP (mmHg)	83 ± 9	81 ± 9	0.011
Heart Rate (bpm)	69 ± 11	66 ± 11	0.004
aPWV (m/s)	6.76 ± 0.65	4.53 ± 0.33	<0.001
Total cholesterol (mmol/l)	4.34 ± 0.9	3.88 ± 0.8	<0.001
HDL (mmol/l)	1.45 ± 0.4	1.42 ± 0.4	ns
LDL (mmol/l)	2.4 ± 0.9	2.1 ± 0.7	<0.001
Triglycerides (mmol/l)	1.16 ± 0.7	0.91 ± 0.6	<0.001
Glucose (mmol/l)	4.88 ± 1.6	4.77 ± 0.70	ns

Data presented as mean ± SD. ns = Not significant.

BMI- Body mass index; SBP-systolic blood pressure; DBP-diastolic blood pressure; MAP-mean arterial pressure; HDL-high density lipoprotein; LDL-low density lipoprotein; aPWV-aortic pulse wave velocity.

*Refers to Top and Bottom deciles of the cohort.

**Figure 2:**
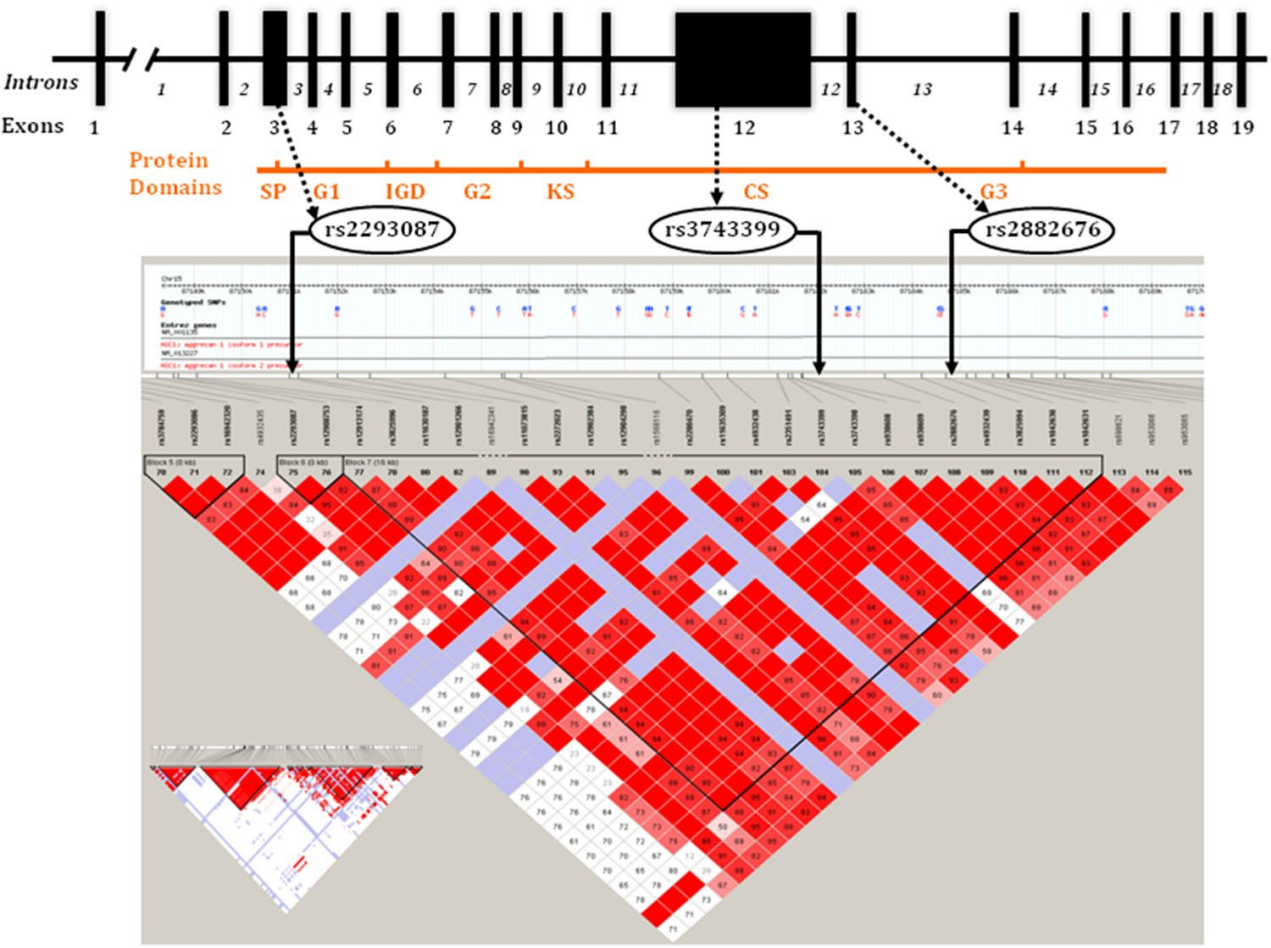
*ACAN* Gene. Genomic map and protein domains with linkage disequilibrium (LD) plot and tagSNPs genotyped.

**Figure 3:**
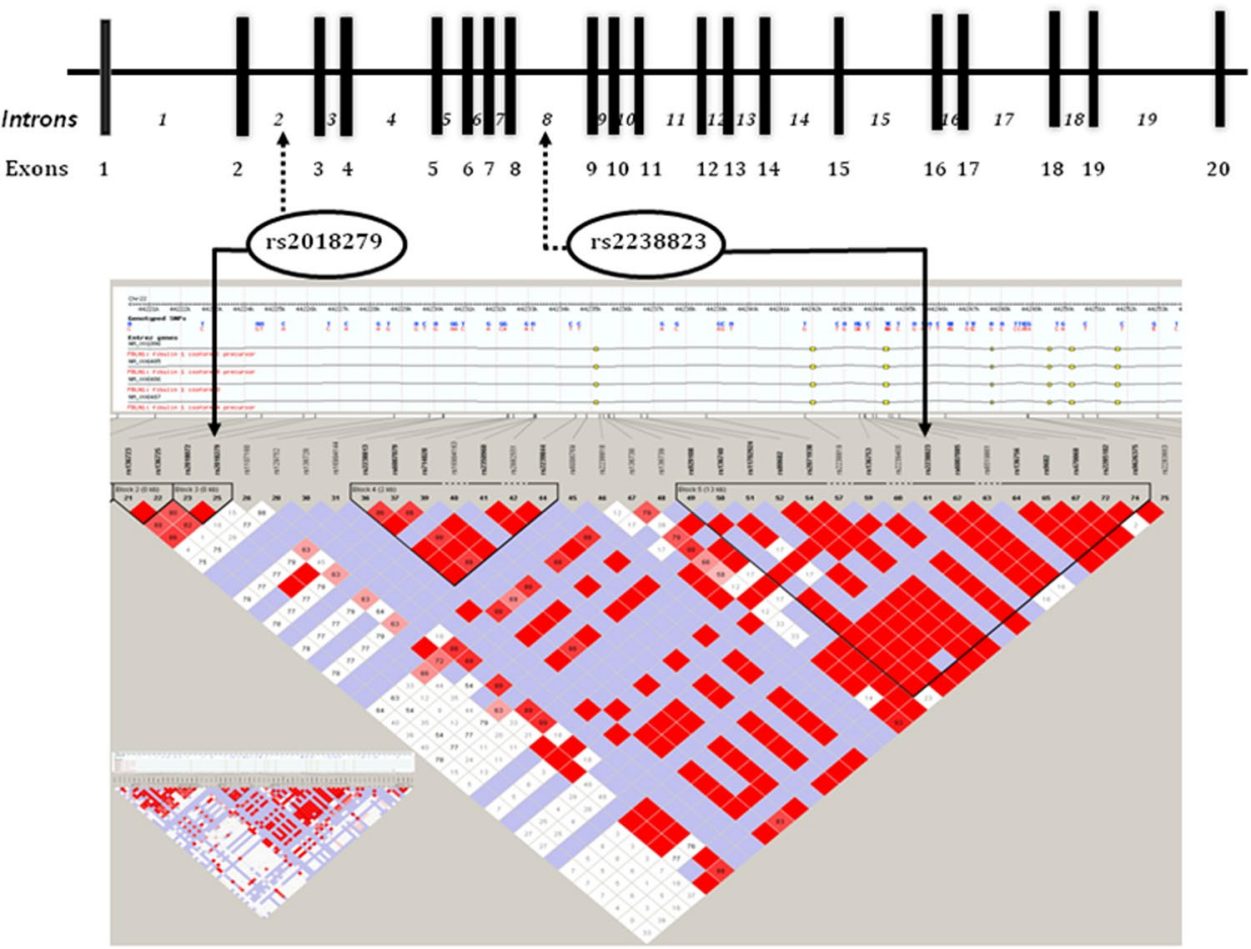
*FBLN*-1 Gene. Genomic map with linkage disequilibrium (LD) plot showing tagSNPs genotyped.

Of the 384 tagSNPs genotyped (Supplementary Table 2), we identified 35 SNPs in 20 genes that significantly associated with aPWV with an unadjusted p-value of <0.05. Of these, 12 tagSNPs in 9 genes (Aggrecan (ACAN), Erythrocyte membrane protein band 4.1-like 2 (EPB41L2), Fibulin-1 (FBLN-1), Fibrinogen (FBG), Interleukin-18 (IL-18), Integrin, alpha6 (ITAG6), Integrin, beta 3 (ITGB3), Matrix metalloproteinase-3 (MMP-3), and Nitric oxide synthase (NOS)), remained significant with a Bonferroni-adjusted p-value of <0.00013 (Supplementary Table 3). We selected five SNPs tagging the two extracellular matrix (ECM) proteins, namely Aggrecan and Fibulin-1, as the most plausible biological candidate molecules for further studies, as these two genes were most up-regulated and associated with aPWV in a previous gene profiling study^4^. Three SNPs in *ACAN* that tagged adjacent LD blocks (chromosome 15; rs2882676A/C in exon 13, rs2293087T/G in intron 3, rs3743399A/G in exon 12; Fig. 2), and two SNPs in *FBLN-1* (chromosome 22; rs2018279A/T in intron 2, rs2238823A/G in intron 8; Fig. 3) correlated significantly with aPWV in subjects pre-selected for extremes of aPWV values.

### Association of *ACAN* and *FBLN-1* Polymorphisms with aPWV

**Figure 4:**
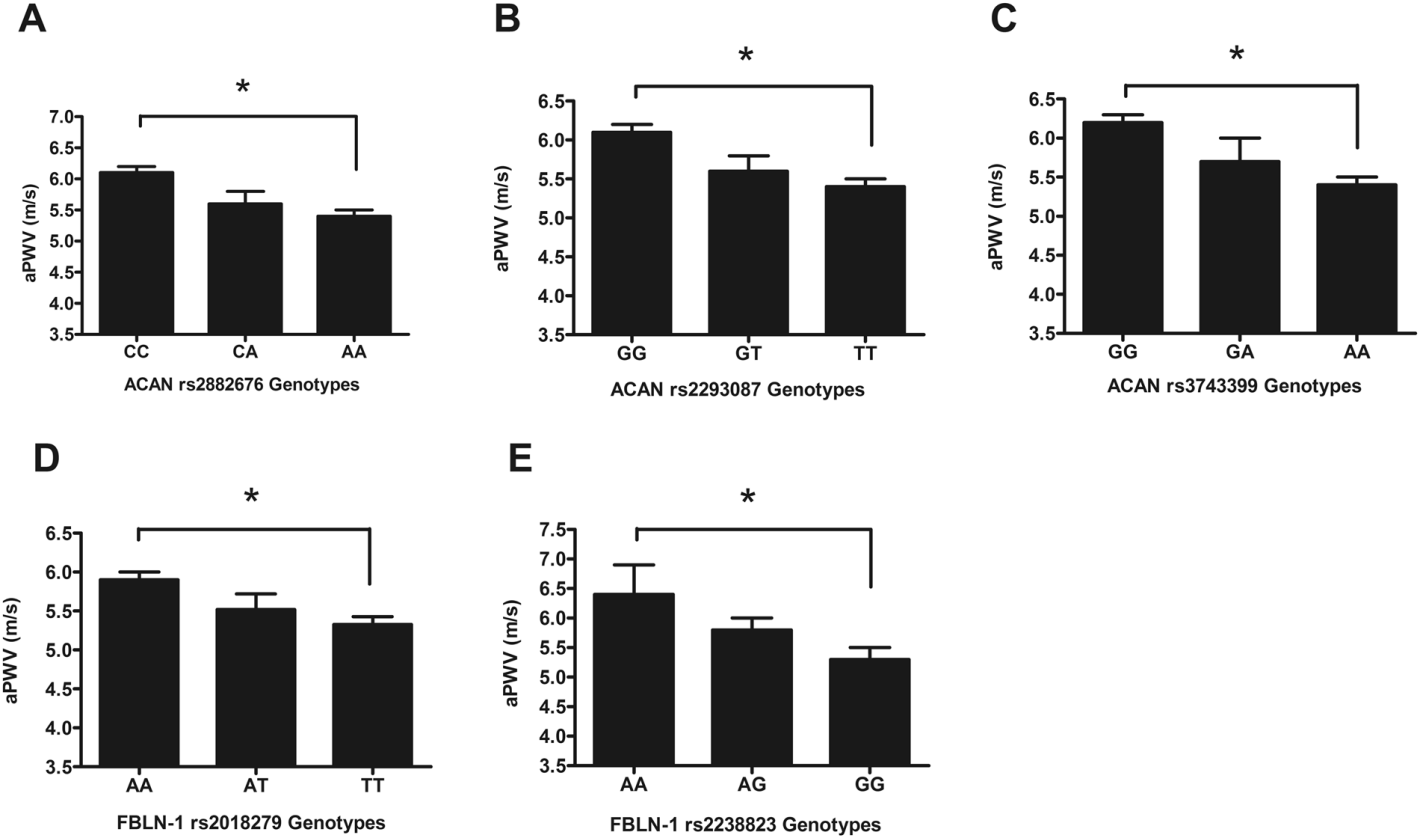
The aPWV is associated with *ACAN* and *FBLN*-1 gene polymorphisms. There is a significant allele dose-effect for each of the *ACAN* and *FBLN-1* SNPs, with homozygotes for CC (**A**), GG (**B**), GG (**C**), AA (**D**) and AA (**E**) having the largest aPWV. ^*^P < 0.001.

In the discovery cohort, aPWV associated significantly with all three *ACAN* polymorphisms. Average aPWV values were significantly higher in subjects carrying the rs2882676/CC, rs2293087/GG, and rs3743399/GG alleles, and a significant allele dose-effect was observed for all three polymorphisms (Fig. 4A–C). Since rs2882676/CC, rs2293087/GG, rs3743399/GG homozygotes had significantly higher aPWV, a stepwise multiple linear regression analysis was performed with additive allele dose models including the main known confounders for velocity (age, mean arterial pressure, heart rate). This analysis showed that the rs2882676/C and rs2293087/G alleles independently predicted aPWV with an adjusted R^2^ of approximately 40% (p < 0.001) for the model, but the rs3743399 polymorphism did not (Table 2).

**Table 2:** ACAN and FBLN-1 gene polymorphisms independently associate with aPWV.

Model Parameters	Standardised coefficient (Beta)	Significance level (p)	R Square change (%)
**Dependent variable: aPWV**			
*ACAN Gene Polymorphisms*			
MAP	0.51	<0.001	36
Age	0.17	<0.001	2
Heart rate	0.13	<0.001	1
rs2882676 A/C	0.11	0.003	1
*Adjusted R*^*2*^ *value* = *0*.*40; F* = *74*.*670; p* < *0.001*			
MAP	0.50	<0.001	36
Age	0.18	<0.001	2
Heart rate	0.16	<0.001	1
rs2293087 T/G	0.09	0.027	1
*Adjusted R*^2^ *value* = *0*.*41; F* = *66*.*715; p* < *0*.*001*			
MAP	0.52	<0.001	36
Age	0.17	<0.001	2
Heart rate	0.12	0.003	1
rs3743399 G/A	0.05	0.151	—
*Adjusted R*^2^ *value* = *0*.*39; F* = *72*.*336; p* < *0.001*			
*FBLN-1 Gene Polymorphisms*			
MAP	0.51	<0.001	36
Age	0.16	<0.001	2
Heart rate	0.13	<0.001	1
rs2018279 A/T	0.09	0.003	1
*Adjusted R*^2^ *value* = *0*.*40; F* = *73*.*250; p* < *0*.*001*			
MAP	0.52	<0.001	36
Age	0.17	<0.001	2
Heart rate	0.12	0.002	1
rs2238823 A/G	0.08	0.003	1
*Adjusted R*^2^ *value* = *0*.*40; F* = *70*.*204; p* < *0*.*001*			

*FBLN-1* polymorphisms also correlated significantly with aPWV in the ENIGMA discovery cohort (Fig. 4D,E). Mean aPWV values were lower in rs2018279/TT and rs2238823/GG allele carriers compared to their respective homozygotes. Again, assuming an additive inheritance pattern, stepwise regression analysis was performed for aPWV adjusting for main confounding variables, which confirmed that both polymorphisms independently predicted aPWV (adjusted R^2^ of 40%, p < 0.001, Table 2).

Similar SNP trends and associations were observed for each of the *ACAN* and *FBLN-1* SNPs in the replication ENIGMA subset and in the combined dataset. But as expected, the discovery cohort with extreme aPWV values showed bigger genotypic differences for aPWV compared to the replication subset with mid-range values (Fig. 1) and the combined dataset (Supplementary Table 3).

### Studies in Human Aortic Tissue Samples

To further explore our SNP findings from the ENIGMA cohort, we genotyped four tagSNPs that predicted aPWV in the human donor aortic tissue samples (age range 17–83 years) collected through our NHSBT transplant coordinators in Cambridge. We also measured *ACAN* mRNA levels for rs2882676 that best predicted aPWV (Table 2) to see if it was an eSNP.

### *ACAN* and *FBLN-1* SNPs, mRNA and Protein Expression in the Aorta

**Figure 5:**
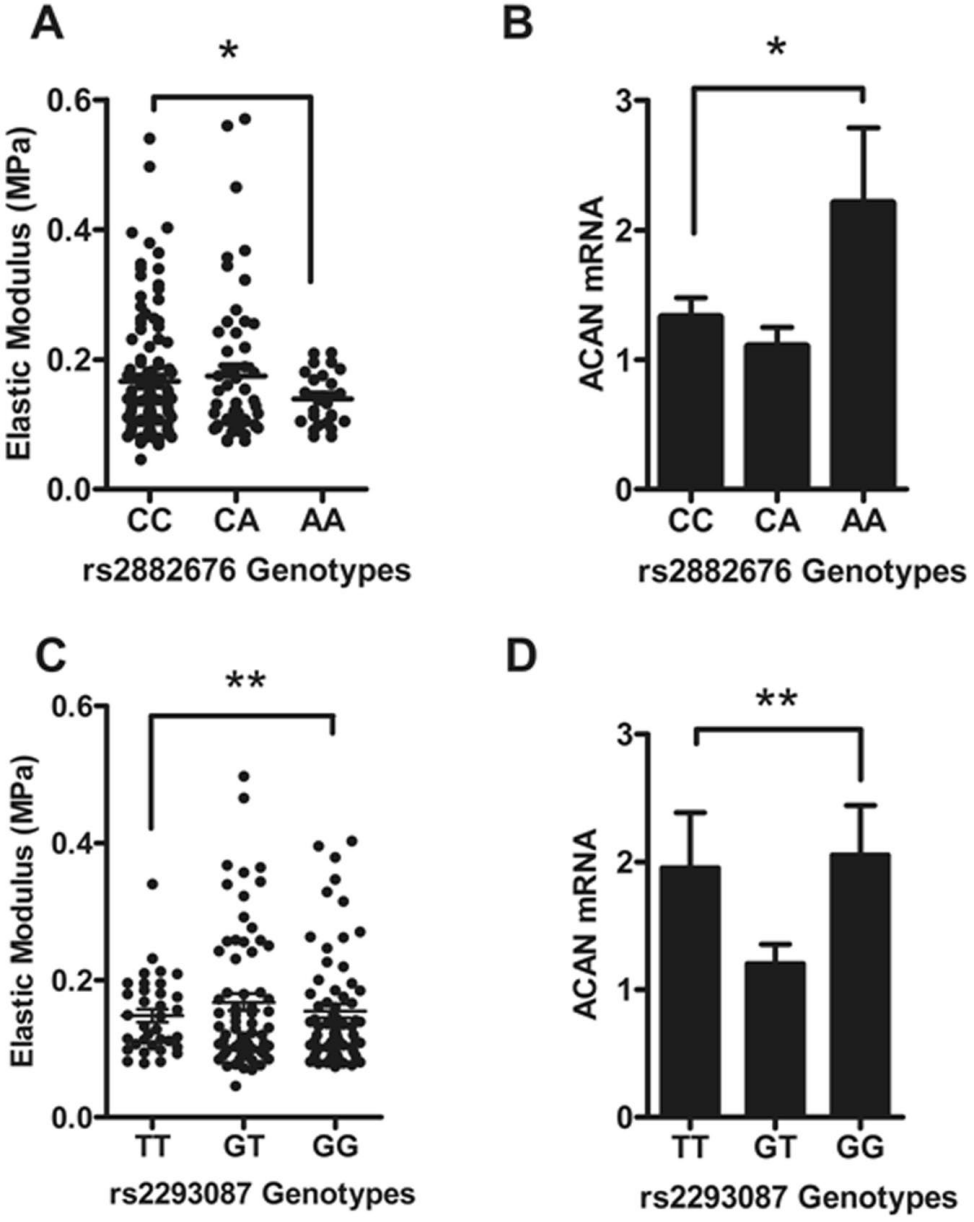
*ACAN* gene polymorphisms associate with aortic stiffness and gene expression. (**A)**, **(C**) Elastic modulus compared to rs2882676 and rs2293087 genotypes. CC and GG donors had stiffer arteries compared to AA and TT ones (^*^P = 0.024, ^**^P > 0.05). (**B**), (**D**) Transcript expression pattern of rs2882676 and rs2293087 across the three genotypes (^*^P = 0.04, ^**^P > 0.05). Bar graph represents the mean ± SEM of ACAN transcript, normalized against GAPDH.

We next looked for correlation of SNP genotypes with an *ex vivo* measurement of aortic stiffness. The ‘elastic modulus’ (EM) directly correlates to PWV, and all four polymorphisms showed similar trends in the human aortic rings as aPWV did in the ENIGMA cohort. However, this trend was only significant for the rs2882676 exon 13 *ACAN* SNP, where donors homozygous for the rs2882676/C allele had higher EM values indicating stiffer arteries (Fig. 5A). The CC allele carriers for this SNP also showed significantly lower (1.7-fold) *ACAN* mRNA expression as compared to those homozygous for A allele (Fig. 5B), confirming that this SNP was a functional eSNP.

**Figure 6:**
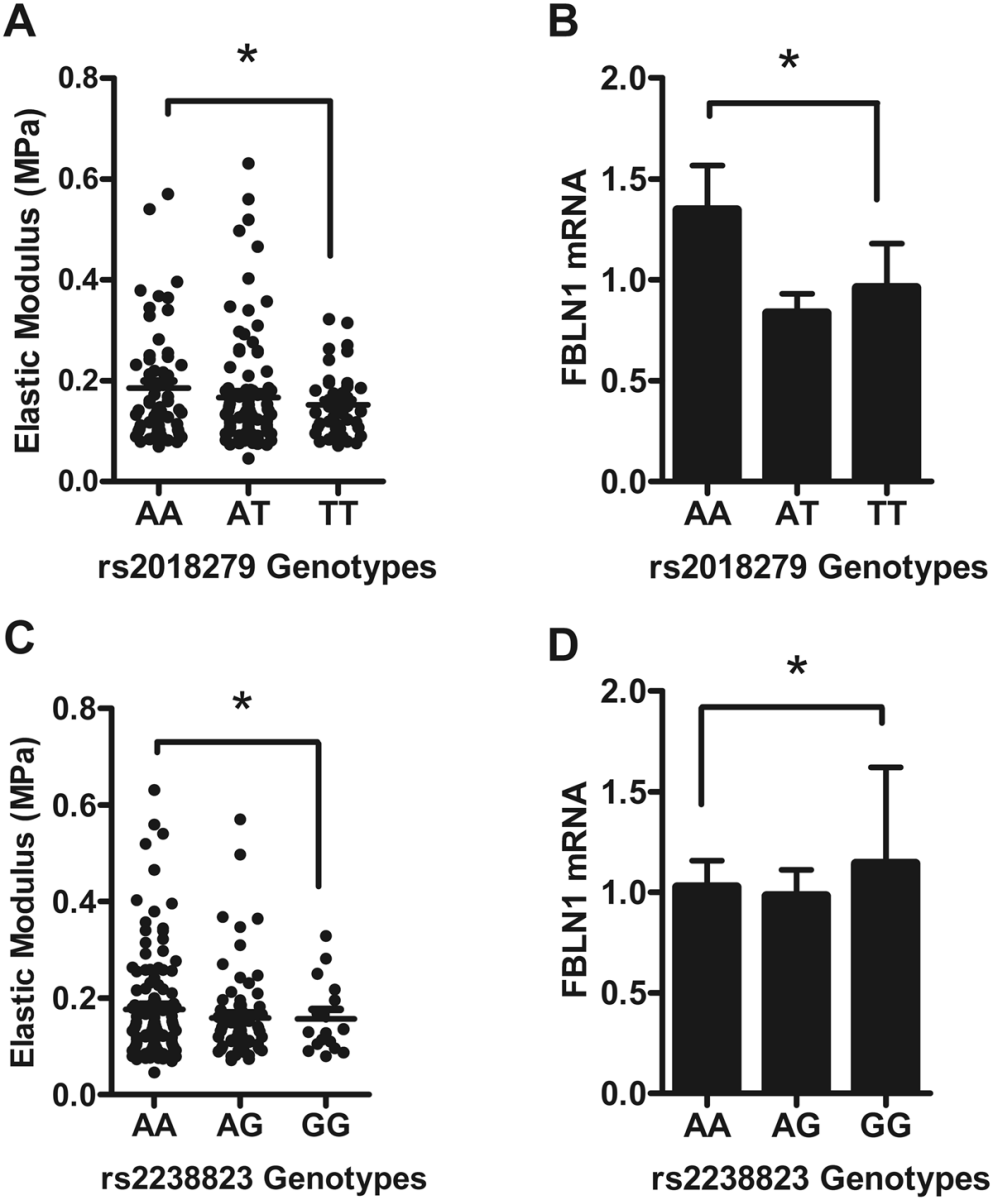
*FBLN-1* gene polymorphisms relationship with aortic stiffness and gene expression. (**A**), (**C**) Elastic modulus compared to rs2018279 and rs2238823 genotypes. (**B**), (**D**) Transcript expression level of rs2018279 and rs2238823 polymorphisms. Bar graph represents the mean ± SEM of *FBLN-1* gene expression normalized against GAPDH. *P > 0.05.

Conversely, donors with the *ACAN* rs2293087 SNP or the *FBLN-1* SNPs, rs2018279 and rs2238823 genotype, did not have significantly different EM or mRNA levels compared to their respective homozygotes (Figs 5C,D and 6).

### Association of *ACAN* and *FBLN-1* Gene Expression with EM in Aortic Rings

**Figure 7:**
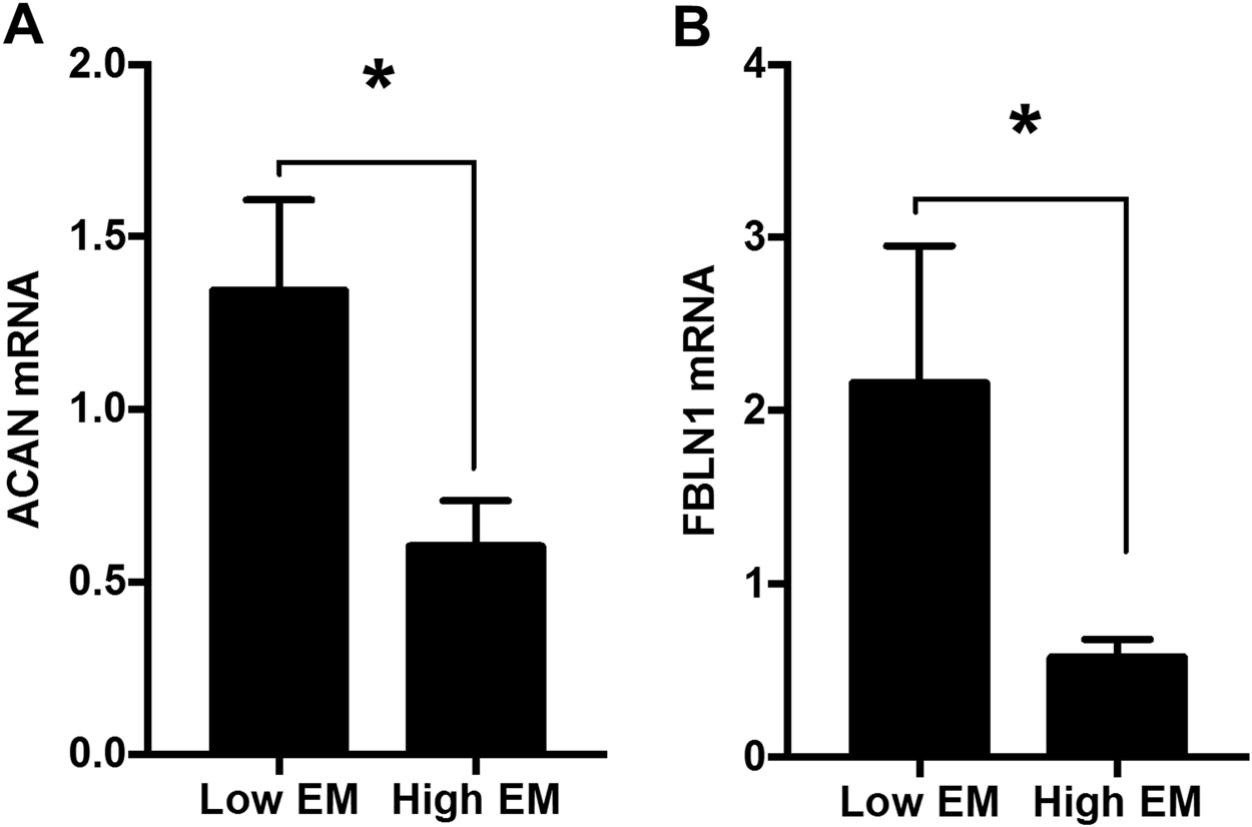
Transcript levels. (**A**) A*CAN* and (**B**) *FBLN-1* in donor aortas (n = 52) that showed either low or high elastic modulus (EM) *ex vivo*. Bars represent mean ± SEM of *ACAN* and *FBLN-1* gene expression normalized against GAPDH. *P < 0.05.

To further define the functional role of *ACAN* and *FBLN-1*, we looked at transcript abundance in the extremes from the EM distribution i.e. top and bottom 15% respectively (low EM and high EM). Donors in the stiffer high EM group were older, and had higher systolic blood pressure compared to the low EM group (Table 3). According to the donor records, less than 10.6% were on CVD drugs. The high EM had a significantly lower expression of *ACAN* mRNA (2.3-fold) compared to the low EM group (Fig. 7A), which persisted after correcting for age as a confounder (ANCOVA, p < 0.05). The high EM donor aortas also showed significantly lower *FBLN-1* transcript levels (3.8-fold) compared to their low EM counterparts (Fig. 7B). Again the difference held after adjusting for age (ANCOVA, p = 0.01).

**Table 3:** Baseline characteristics of aortic tissue donors.

Parameters	Low EM* (n = 32)	High EM*(n = 28)	Significance level (p)
Age (years)	51 ± 11	66 ± 14	<0.001
Male/Female (n)	17/15	18/10	ns
Elastic Modulus (MPa)	0.08 ± 0.01	0.37 ± 0.10	<0.001
BMI (kg/m^2^)	28 ± 5	28 ± 6	ns
SBP (mmHg)	119 ± 25	130 ± 24	ns
DBP (mmHg)	69 ± 15	64 ± 13	ns
Serum Creatinine at retrieval (µmol/l)	129.2 ± 88.2	111.5 ± 69.1	ns
Glucose (mmol/l)	9.37 ± 4.09	9.47 ± 3.84	ns
Non-Smokers (n)	10	12	ns
Alcohol-Non drinkers (n)	15	12	ns
Hypertension (n)	10	14	—
Diabetes (n)	4	3	—
CVD (n)	2	5	—

Data presented as mean ± SD. ns = Not significant.

BMI- Body mass index; SBP-systolic blood pressure; DBP-diastolic blood pressure.

^*^Refers to the Top and Bottom 15% of the donors.

### Protein and Tissue Distribution of *ACAN* and *FBLN*-1 with Age and EM

**Figure 8:**
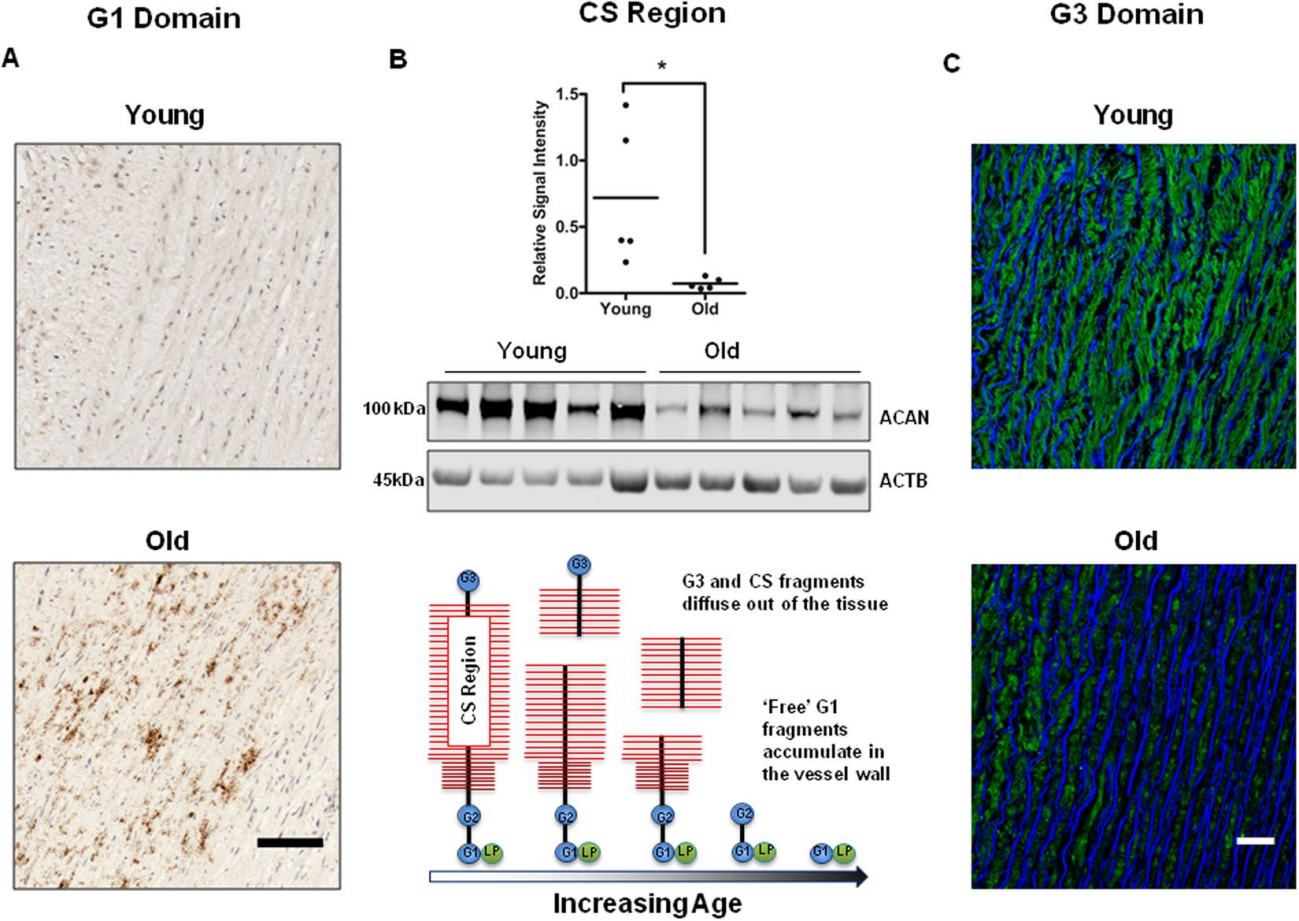
Aggrecan tissue and protein distribution in young (<32 y) versus older (**>**60 y) aortic tissue donors. (**A**) Representative sections showing immunohistochemical staining for the G1-G2 domain of aggrecan in the medial layer. Scale bar, 50 μm. (**B**) Top panel: Quantification plot shows aggrecan protein levels are down-regulated in older donors as compared to younger donors. Each dot on the scatter plot exemplifies the signal intensities of individual donor samples. Bars represent mean signal intensity normalized against β-actin. *P = 0.026. Middle panel: Representative western blots showing the expression of the chondroitin-sulfate (CS) attachment region of aggrecan in young versus older donors. Bottom panel: Schematic diagram illustrates the degradation of C-terminal aggrecan fragments and their diffusion out of the vessel wall with increasing age, accompanied by an accumulation of G1 fragments. CS, chondroitin-sulfate; LP, link protein. (**C**) Representative immunofluorescent staining of aortic tissue shows uniform expression of aggrecan G3 domain (green) across the vessel wall of younger donors that is largely absent from older ones. Scale bar, 50 μm.

Many ECM proteins including aggrecan are altered or degraded with age. We therefore, explored aggrecan protein expression patterns in the aortic wall using antibodies that recognized epitopes on the three main domains. Namely, the N-terminal G1-G2 domain, the chondroitin-sulphate (CS) middle region and the C-terminal G3 domain (see schema in Fig. 8). Immunohistochemical staining showed that aggrecan G1-G2 and G3 domains were present throughout the vessel wall layers of young subjects (Fig. 8A–C). However, in older subjects, a striking deposition of G1-G2 domain staining was seen across the medial layer with a marked loss of immunofluorescent intensity for G3 domain staining (Fig. 8C). This indicated an age-dependent loss of the C-terminals from aggrecan and accumulation of N-terminal fragments in the aortic wall, which mirrors the changes reported in joint cartilage with age^13^. These CS fragments are necessary for viscoelasticity and we confirmed their loss by western blotting aortic wall homogenates. This confirmed a 3.5-fold reduction (Fig. 8B) in immunoblottable CS in the older group (n = 5) compared to the younger group (n = 5).

**Figure 9:**
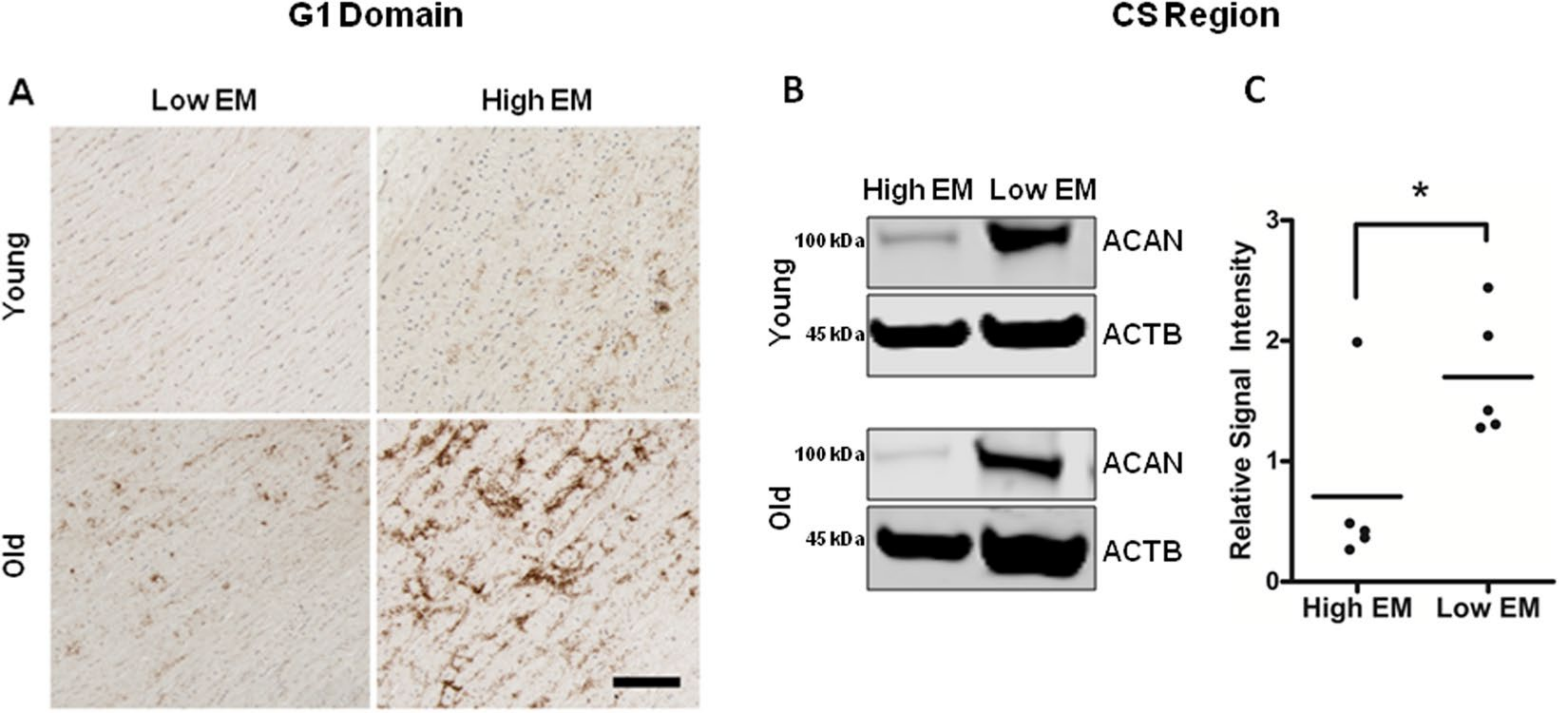
Aggrecan protein domain distribution in young versus old aortas and in high versus low EM donors. (**A**) Representative immunohistochemical staining for G1 domain comparing young (<32 y) versus old donors (>60 y), and in aortas with low and high EM. (**B**), (**C**) Representative western blots showing quantitative analysis of aggrecan-CS signal intensities, normalized against β-actin in independent young and old donor samples with high EM (n = 5, 2 young and 3 old) and low EM (n = 5, 2 young and 3 old) values. Each dot on the scatter plot exemplifies the signal intensities of individual donor samples. Solid bars are the mean signal intensity in the plot. *P < 0.05.

**Figure 10:**
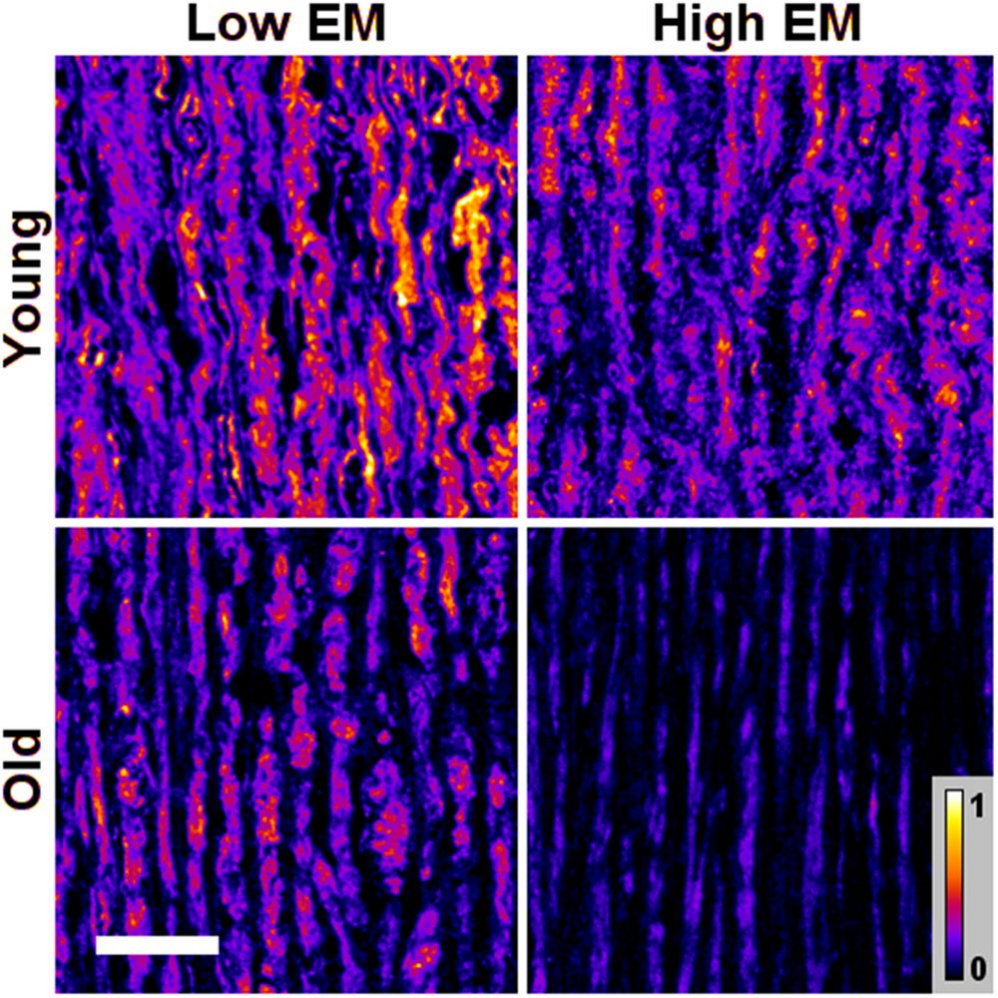
Heat-map distribution of aggrecan protein G3 domain immunostaining in young (<32 y) versus old donors (>60 y), and in low versus high elastic modulus donor aortas. Representative immunofluorescence staining shows higher G3 deposits in young aortas with low EM compared to high EM samples; and in old aortas with low EM compared to high EM group. Heat map scale bar 0 to 1 demonstrates the aggrecan protein distribution and intensity in each sample and in each group. Scale bar, 50 μm.

**Figure 11:**
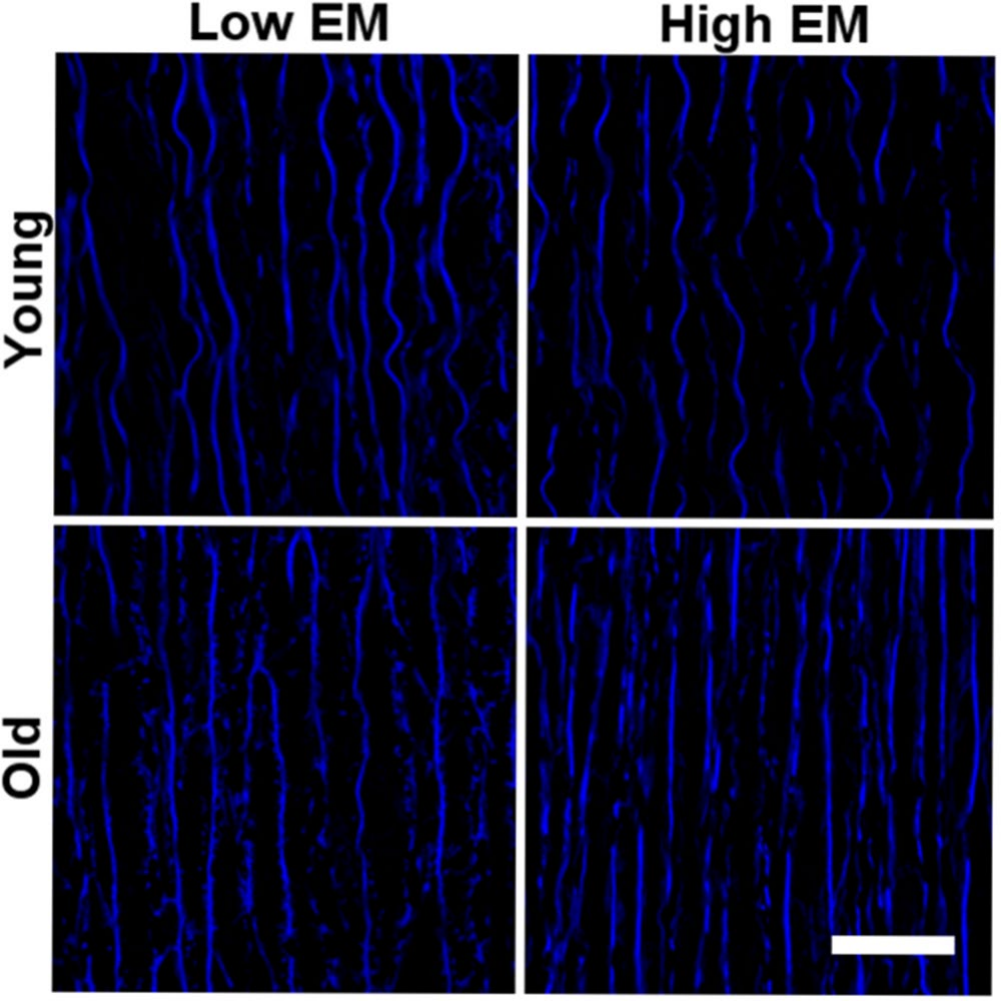
Distribution of elastin fibers in young (<32 y) versus old (>60 y), and in low versus high EM donor aortas. With aging and increased stiffness, elastic fibers are frayed and fragmented. Scale bar, 50 μm.

We next compared aggrecan protein expression patterns in independent young and old donor aorta samples based on their *ex vivo* elasticity (top and bottom 15% of EM values). Interestingly, we found that G1-G2 staining pattern was more typical of the older aortas (p < 0.05, Fig. 9A), and the stiff young donor aortas (high EM) had the lowest expression of CS region as compared to the elastic aortas ((low EM), Fig. 9B,C)). Young distensible aortas (low EM) also expressed strikingly more G3 compared to both older and stiffer aortas (high EM). This preservation of intact aggrecan molecules was best seen using a heat map (Fig. 10). Of note, the G3 staining inversely related to aortic elastin disorganisation (Fig. 11).

**Figure 12:**
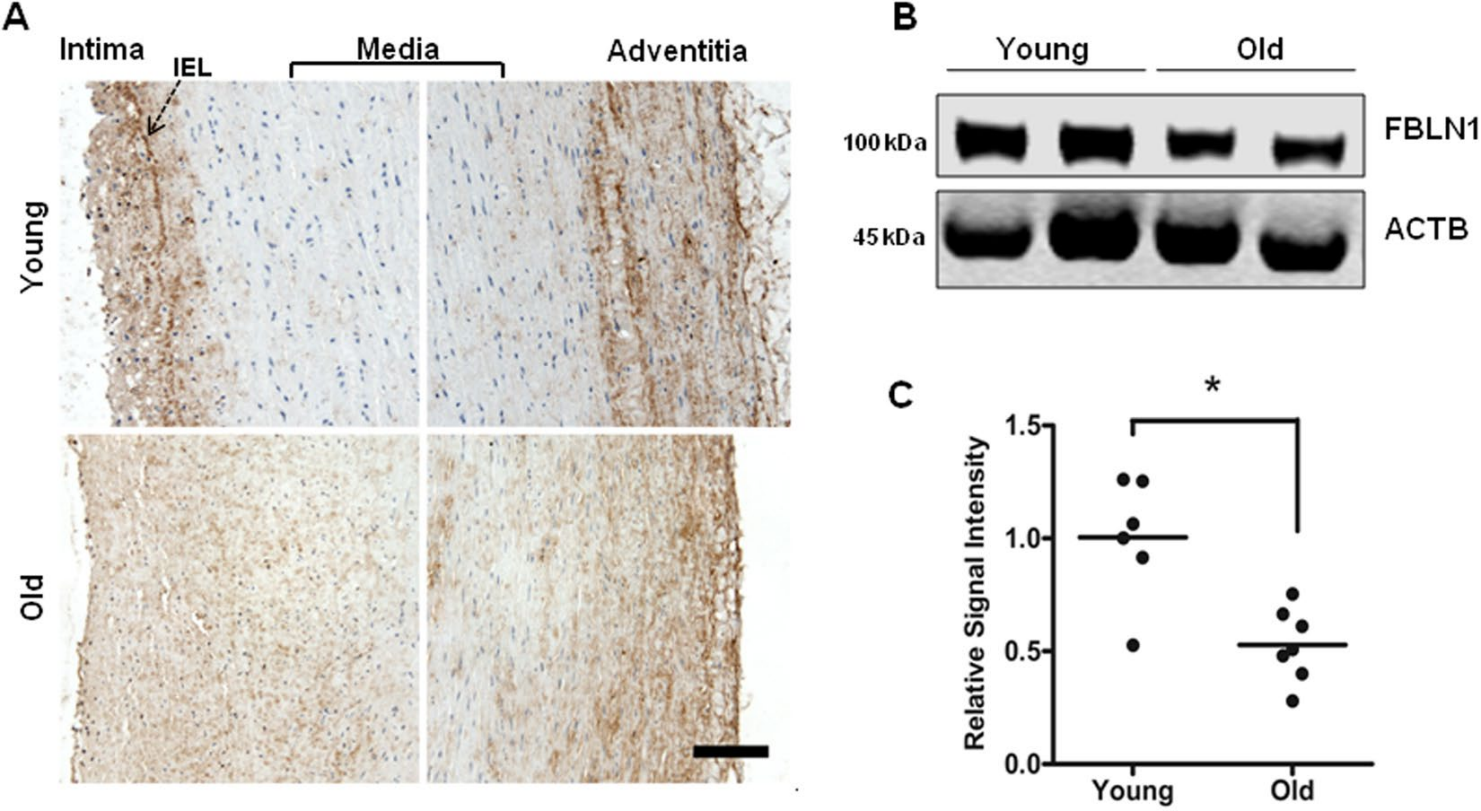
Fibulin-1 tissue and protein distribution in young versus older donor aortas. (**A**) Representative immunohistochemical staining showing the pattern of FBLN-1 distribution in aortic sections from a young (<32 y) versus an old donor (>60 y). Fibulin-1 is expressed throughout the vessel wall but appears denser in the intima and adventitial side of media in young aorta compared to the more even distribution in the older aorta. Scale bar, 50 μm. (**B**) Representative western blot comparing fibulin-1 protein expression in young and old donors. (**C**) Quantitative analysis of fibulin-1 signal intensities, normalized against β-Actin in young (n = 6) versus old donor tissues (n = 7). Each dot on the scatter plot exemplifies the signal intensities of individual donor samples. Solid bar is the mean intensity. *P = 0.008.

**Figure 13:**
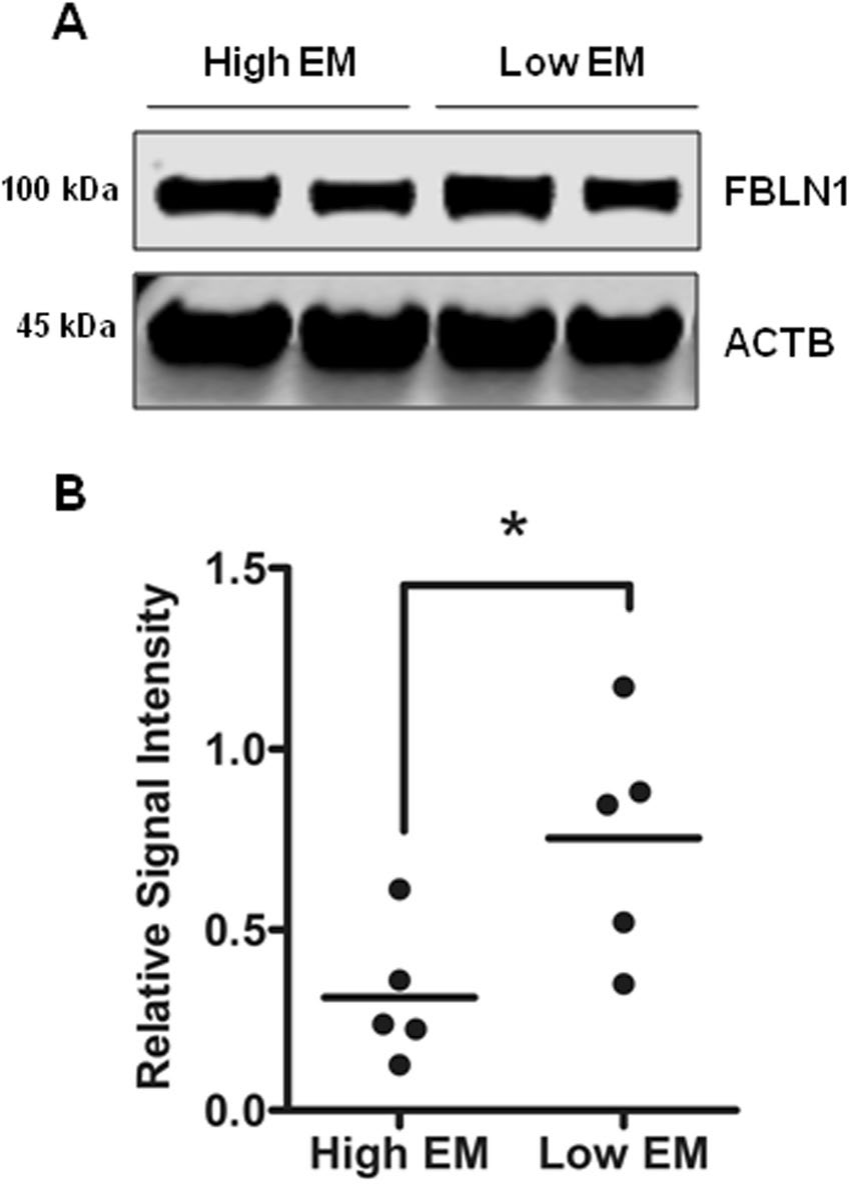
Fibulin-1 protein distribution in aortas with low and high EM. (**A**) Immunoblotting shows the fibulin-1 protein expression pattern in the high and low EM groups. (**B**) Quantification plot shows fibulin-1 protein levels are lower in high EM group as compared to low EM group (n = 5 in each group). Each dot on the scatter plot exemplifies the signal intensities of individual donor samples. Bars represent mean signal intensity normalized against β-actin. *P = 0.029.

When fibulin-1 protein expression patterns were examined in tissues from younger (<32 yrs) and older (>60 yrs) donor aortas, the protein was evident across all layers of the vessel wall in both age groups (Fig. 12A). However, fibulin-1 expression was more prominent in the tunica intima and adventitial side of media in the younger donors (Fig. 12A). Immunoblotting of whole aortic lysates also showed that total fibulin-1 was actually higher in young donors versus old donors (p = 0.008, Fig. 12B,C), indicating age-related changes. Moreover, when the difference in fibulin-1 protein expression between low and high EM aortic homogenates was assessed, we found significantly reduced fibulin-1 protein levels in the high EM group as compared to the low EM group (p = 0.029, Fig. 13A,B), signifying fibulin-1 protein also alters with increased stiffness.

## Discussion

The extracellular matrix (ECM) plays a central role in both the age-related remodelling of the vessel wall and its response to hypertension or injury. As proteoglycans are key components of the ECM and hence wall stiffness^14^, we focused on *ACAN* and *FBLN-1* amongst the hits from our discovery study. Aggrecan is a protein best understood from its role in joint cartilage where its domain structure and function has been extensively investigated; especially its contribution to the viscoelastic properties of cartilage^15^. Mutations in *ACAN* have been linked to monogenic human skeletal disorders such as osteochondritis dissectans (OMIM 165800)^16^ and spondyloepiphyseal dysplasia (OMIM 612813), and VNTR polymorphisms with premature degenerative lumbar disk degeneration^17,18^ and osteoarthritis itself^19,20^. We found the exonic (rs2882676/C) and intronic (rs2293087/G) tagSNPs associate significantly with higher aPWV values in our young subjects. Additionally, we found the exonic rs2882676 SNP modulates the elastic modulus (EM), an *ex vivo* measurement of stiffness in human donor aortic tissues. The tagSNPs we used were on different domains: the intronic rs2293087 was in the G1 region; the exonic synonymous rs3743399 and non-synonymous rs2882676 were in chondroitin sulphate (CS) attachment and G3 regions respectively. The latter coding for EGF-like domains involved in the Ca^2+^ binding process^15^. Prior to this work a biological role for these domains in the vasculature was not suggested, but aggrecan has now been linked with vascular dementia, and increased aortic stiffness is associated with small vessel disease in the brain^21^. More recently, Suna *et al*. demonstrated the role of aggrecan and aggrecanase activity in a pig model of the injury response to intracoronary stenting^22^. In a separate study, the same authors also showed that aggrecan protein was more abundant in human coronary artery and thoracic aorta versus veins, and showed that the cells responsible for aggrecan and aggrecanase activity expression were in fact the vascular smooth muscle cells. This new data strongly implicates a role for aggrecan in vascular plasticity and arterial remodelling.

Carriage of the *ACAN* rs2882676/C allele was an independent predictor of aortic stiffness (aPWV) in this study even after removing the influence of typical confounding factors. This SNP changes the amino acid from glutamine to alanine at position 1508 (E1508A), and non-synonymous SNPs are often assumed to be functional especially in cases where the substitution changes resides in an important domain or motif in a protein^23^. We do not know if this is the case for the E1508A substitution in aggrecan, but the same SNP was identified in a GWAS as a risk allele for late-onset Alzheimer’s disease^24^. We have also shown that it affects *ACAN* transcript levels and associates with *ex vivo* aortic stiffness.

The N-terminal G1 domain of aggrecan interacts with hyaluronan and a link protein, forming a stable complex anchoring the aggrecan molecule to the tissue. Glycosaminoglycans (GAGs) bind to the large central G2 domain of aggrecan that typically carries keratin sulphate (KS) and >100 chondroitin sulphate chains per molecule. Negative charge provided by these sulphate groups attracts counter ions and draws water into the extracellular matrix. Aggrecan thus gives viscoelasticity and load-bearing properties to tissues in which it is expressed. So heterogeneous distribution of proteoglycans such as aggrecan across the aortic wall is probably crucial in regulating the residual stress of the aorta^25^. Aggrecan structure and expression does not remain constant at other sites throughout life. For instance, in human articular cartilage, intervertebral disc and sclera, aggrecan undergoes an age-dependent proteolysis resulting in a progressive loss of its C-terminal and accumulation of N-terminal G1 fragments^13,26–28^. For the first time, we show that a similar age-dependent process of aggrecan degradation occurs in the human aortic vessel wall. We also show that *ACAN* transcript abundance declines with age and is significantly lower in individuals with stiffer aortas (high EM). In line with our findings, transcript abundance of *ACAN* and other proteoglycan core proteins falls in the aortas of adult mice compared to late stage embryos^29^. The total GAG content in human thoracic aorta also decreases after the age of 40^30^ and is lower in non-atherosclerotic ascending aortas of old subjects compared to younger ones^31^. Intriguingly, Manley *et al*. observed a higher number of amino-acids associated with CS chains in old aortic samples compared to younger ones^31^: approximately 2 serine molecules per GAG chain in older subjects and 1 serine molecule per GAG chain in younger subjects. We suspect that the fragmentation of aortic aggrecan exposes additional sites on CS chains for attachment with serine residues. Conversely, our data conflicts with previous findings by Durier *et al*.^4^, who reported increased *ACAN* transcript abundance in association with increased aPWV. This discrepancy may reflect their very small sample size (n = 9) and heterogeneity of the samples taken from coronary artery bypass grafting patients (>50 yrs).

**Figure 14:**
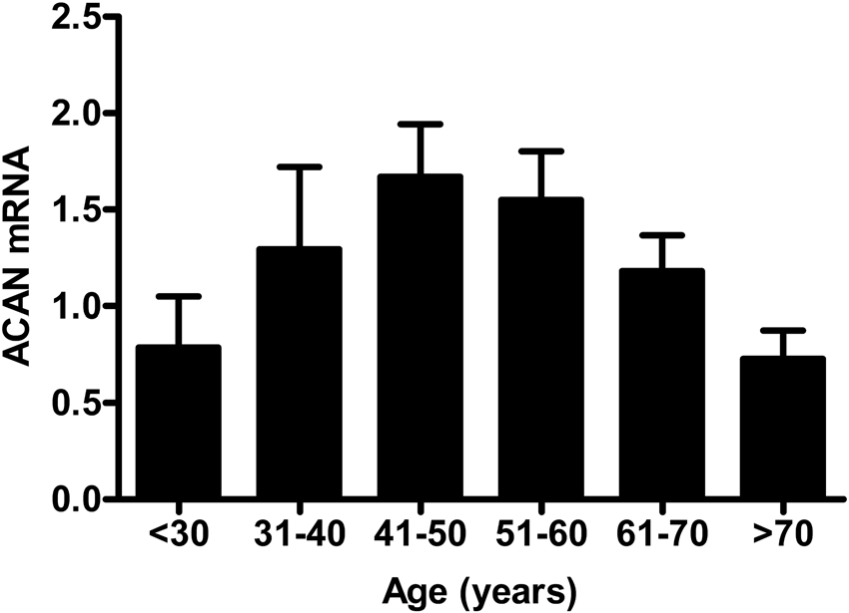
Transcript expression pattern of *ACAN* across age decades. Bars represent mean ± SEM normalized against GAPDH.

The age-dependent loss of aortic aggrecan may partly be explained by reduced synthesis of the protein (from reduced transcript levels), as suggested by a notable decline in transcript levels after the fifth decade (Fig. 14). However, the accumulation of G1 fragments in the medial layer of older subjects implicates post-translational modifications of ECM components. Aggrecan is degraded by a number of proteases including disintegrin and metalloproteinase with thrombospondin motifs (ADAMST-4,5), matrix metalloproteinases (MMP-1,2,3,8,9,13), cathepsins and calpains^32^. The CS and inter-globular domain regions are particularly susceptible to proteolytic cleavage^27^. In the aorta, aggrecan degradation is likely to be due to increase in the activity and expression of aortic MMP-2, MMP-9 and Calpain-1 with advancing age^33–37^, whilst increased ADAMTS levels promote thoracic aortic aneurysm progression^38^.

The proteolytic degradation of aggrecan and reduced *ACAN* expression could contribute to the stiffening of aorta in several ways. Firstly, GAGs such as aggrecan, are involved in the load-bearing properties of tissues. To achieve this, aggrecan forms large complexes with other matrix proteins such as Fibulin, Tenascin and Lumican, which are restrained in the tissue by a scaffold of collagen fibrils that contributes to the ECM organization^28,39,40^. Loss of aggrecan functionality could therefore impact on viscoelasticity, medial ECM disorganization and collagen fibril redistribution to modulate vessel stiffness. Secondly, the concomitant accumulation of G1 fragments in the medial layer may further promote its degeneration and pathological ECM remodelling. Lastly, the degradation of aggrecan weakens its intrinsic ability to suppress calcium phosphate formation^41^. It is highly likely that aortic wall mineralization, another driver of aortic stiffness, is facilitated by aggrecan fragmentation and degradation^42^.

The fate of the cleaved G3 and CS fragments in the aorta remains to be established but are potentially important. They may be released into the circulation, where they could provide a novel biomarker for aortic stiffness. Although this may be difficult to distinguish from the signal caused by the fragments exiting joint cartilage with age. This hypothesis is supported by the presence of GAGs in old aortas that were digested *ex vivo*^31^. A better understanding of the precise mechanisms regulating aggrecan decline could also identify novel therapeutic targets. These might be aimed at either restoring aortic aggrecan content or preventing its proteolytic degradation. Current strategies targeting aggrecan include the use of aggrecan analogues resistant to proteolysis in articular cartilage repair^43^, aggrecanase inhibitors to improve cardiac function in pressure overload models^44^, and the development of specific ADAMTS-4/5 inhibitors^45,46^. All have undergone clinical trials for osteoarthritis^47^, and could be plausibly repurposed as therapeutic strategies for aortic stiffness.

Fibulin-1 is an ECM protein present throughout the arterial wall, but its expression is highest in the outermost layer of the tunica media in association with external elastic lamina^48,49^. Nevertheless, the clinical significance of fibulin-1 gene (*FBLN-1*) in the vasculature remains unclear. There are, for example, no clear associations of *FBLN-1* with human disease, but there is evidence linking *FBLN-5* with altered biomechanical and microstructural properties in animal models^50^. Besides being an ECM protein, fibulin-1 circulates in high concentrations in plasma and is a cardiovascular disease biomarker reflecting elastolysis^51,52^. It is also associated with heart failure^53^, severe aortic stenosis^54^, vascular calcification^55^, up-regulated in non-atherosclerotic diabetic tissues^51^, and affected by arterial wall mechanics^4^. Moreover, fibulin-1 up-regulation is linked with increased stiffness^56^ and kidney disease^52^, whilst its down-regulation is related to aortic dissection^57,58^. Fibulin-1 levels are also affected by drug interventions, so patients treated with spironolactone show reduced levels in parallel with regression of vascular remodelling^59^.

Given the relation of fibulin-1 with elastic fibres in the vessel wall, it is perhaps not a surprise that we found associations between aPWV and intronic fibulin-1 polymorphisms (rs2018279, rs2238823) in this study. They are also independent predictors of aPWV. The SNPs themselves are not eSNPs, so thay are either affecting expression of other transcripts or in LD with other functional SNPs. Expression of fibulin-1 transcript and protein was down-regulated in donors with stiff aortas and aortas from older donors had significantly lower fibulin-1 protein expression than younger ones. This is the same pattern we saw with aggrecan, but may not be coincidental. Fibulin-1 is a high affinity ligand for the G3 domain of aggrecan^60^, so reduction in fibulin-1 expression may, at least in part, be explained by the loss of aggrecan from the vessel walls. Our data are in accord with the observations found in tissues recovered from acute aortic and Stanford type A dissections, where fibulin-1 was down-regulated^57,58^. Age-related elastin fragmentation and medial degeneration are the hallmarks of aortic stiffness leading to aortic dissection, which presumably explains the parallel loss of fibulin-1. Fibulin-1 is thought to interact with elastin and microfibrils, and therefore plays a role in elastic fibrogenesis contributing to the structural integrity of the vessel wall^48^. Currently, it is not clear whether the observed decline in fibulin-1 expression contributes to the age-related fraying and fragmentation of elastin fibres or is a consequence of it. Further studies are needed to address this key point.

In summary, we demonstrate for the first time that common polymorphisms in the genes for aggrecan and fibulin-1 predict aortic stiffness in young healthy subjects. We also show that stiff young donor aortic tissue has changes in the expression and distribution of these proteins that are more typical of stiff old aortic tissue. So, loss and degradation of these proteins appear to be important drivers for age-related aortic stiffening (ARAS) and modulating these changes could slow or arrest the aortic stiffening process.

## Methods

### Study participants

A total of 2000 subjects who participated in the ENIGMA study between 2002–2007, and a part of the Anglo Cardiff Collaborative Trial (ACCT)^61^, and aged between 18 and 30 years, were studied as part of an investigation into the physiological factors relating to the development of hypertension in young adults. Individuals were selected at random from the Universities of Swansea and Cambridge, UK by advertisement and word of mouth. Subjects from this cohort who were <25 years old were used for the genetic studies. A small number of subjects with systolic and/or diastolic hypertension (SBP/DBP ≥140/90 or diastolic ≥90 mmHg), diabetes or hypercholesterolemia (total cholesterol ≥6.5 mmol/L) were excluded from this age-restricted ENIGMA subset, as were subjects receiving any cardiovascular medication. The Local Research Ethics Committees (LREC/01/203), approved the study, and written informed consent was obtained from all participants. All experiments were performed in accordance with the relevant guidelines and regulations.

All samples and donor data were handled in accordance with the policies and procedures of the Human Tissue Act (UK). The Local and Regional Ethics Committees approved the study (MREC/03/2/074).

### Haemodynamic measurements

All studies were conducted in a quiet temperature-controlled room. Height and weight were recorded, and body mass index was calculated. After 10 minutes supine rest, peripheral blood pressure was recorded in the dominant arm using a validated oscillometric device (HEM-705CP; Omron Corporation). Carotid and femoral artery waveforms were recorded with a high fidelity micromanometer (SPC-301; Millar Instruments) using the SphygmoCor system (AtCor Medical), as previously described^62^. aPWV was calculated from the foot-to-foot delay between carotid and femoral waveforms and body surface measurements that adjusted for parallel transmission in the carotid and aorta by using the suprasternal notch as a fiducial point as described previously. All measurements were made in duplicate, and mean values used in the subsequent analysis.

### Laboratory measurements

Blood samples were drawn, serum and plasma separated and stored at −80 °C. Total cholesterol, triglycerides, and glucose were determined using standard methodology in an accredited laboratory. Genomic DNA (gDNA) was extracted from the venous blood using the commercial kit in accordance with the manufacturers protocol (Invitrogen, Lifetech Biosciences). The quality and quantity of gDNA was determined using Nanospectrophotometer and PicoGreen methods and samples stored at −80 °C freezer until further analysis.

### Genetic association studies in ENIGMA

#### tagSNPs selection

31 genes from gene expression profiling work done by Durier *et al*.^4^ and 22 genes previously associated and/or implicated with aPWV and/or arterial wall properties^5,63^ were investigated (Supplementary Table 1). Details of the genes, SNPs and their selection, and the genotyping criteria followed are described in Supplementary Material. Briefly, the tagSNPs (n = 384) for each gene were selected from the public databases, including the International HapMap project^64^, SeattleSNPs database^65^, and previously published candidate gene/SNP association studies.

#### Primary study

A total of 384 tagSNPs were genotyped in 480 ENIGMA study participants using the custom designed VeraCode GoldenGate^TM^ assays on Illumina BeadArray platform (Illumina Inc., San Diego, USA). Supplementary Table 2 presents detailed information of the 384 tagSNPs. Illumina system uses a high density BeadArray technology in combination with allele specific extension, adapter ligation and amplification assay protocol. The technology uses two alternative oligo probes that bind specifically to one or other of the two possible SNP alleles, and a third oligo probe that is locus specific, downstream to the allele-specific oligo of approximately 1–20 base pairs from the SNP sequence used to query each SNP. Briefly, 5 ul of 50 ng of biotinylated DNA was bound to paramagnetic beads and mixed with a pool of SNP-specific oligonucleotides for annealing. The oligonucleotides that hybridized were then extended and ligated to generate DNA templates which were amplified using universal fluorescently-labeled primers. Single-stranded PCR products were then hybridized to a Sentrix® Array Matrix and the arrays were imaged using a BeadArray Reader Scanner. All samples were genotyped in duplicate in the same array/plate and also a number of samples were repeated in different plates. Genotypes were called using the GenCall data analysis software (Illumina Inc., San Diego, USA). In some SNPs where the genotype clusters were unambiguous, manual calling was performed. Majority of the SNPs genotyped were in Hardy Weinberg Equilibrium (HWE) and the SNP call rate was 99.98%.

#### Genotyping strategy

This was performed in two stages: first, subjects selected according to the top (n = 240) and bottom (n = 240) deciles of aPWV values were genotyped using a custom designed assay (Fig. 1, Supplementary Table 2). This strategy was chosen as risk alleles for stiffness can be identified in the phenotype extremes, and also to reduce the cost of genotyping. In the second stage, SNPs/loci that passed a significant threshold (p < 0.05) were further pruned down with bonferroni correction and only those SNPs that were highly significant (p < 0.00013, Supplementary Table 3), and belonged in particular, to the extracellular matrix (ECM) i.e., Aggrecan and Fibulin-1 genes were selected for genotyping, as these genes were up-regulated and associated with aPWV in the gene profiling work.

### Replication study of *ACAN* and *FBLN-1* polymorphisms

In total, five polymorphisms: three in aggrecan (rs3743399, rs2882676, rs2293087) and two in fibulin-1 (rs2018279, rs2238823) genes were validated in the remainder of the ENIGMA cohort (group with mid-range aPWV values, n = 1233, after excluding participants with aPWV values of <3.5 and missing data) using the ABI Taqman 7500 Sequence Detecting System and SNP genotyping assays (Applied Biosystems, USA).

Briefly, Taqman technology uses two alternative oligomer probes that bind specifically to one or other of the two possible SNP alleles. Briefly, 100 ng of gDNA was mixed with 2x Taqman Universal Master Mix, No AmpErase UNG, labelled probe (FAM and VIC dye-labelled) and MQ H_2_O to a total volume of 15 µL for each sample. The SNP is PCR amplified and during PCR, each probe binds to its target allele and the fluorescent reporter dye (which is different to each probe) is cleaved and released into solution by the 3′→5′ exonuclease activity of the *Taq* polymerase. Reporter dye intensity at the end of PCR reaction was then used to quantify levels of each SNP in the sample. The PCR thermal cycling conditions involved an initial denaturing at 95 °C for 10 minutes, followed by 40 cycles at 95 °C for 15 seconds and 60 °C for 1 minute. Allelic discrimination was carried out by detecting allele specific fluorescence and data was analyzed off-line with the sequence detection software (version 1.9).

### Validation study of ACAN and FBLN-1 proteins in human aortic tissue samples

Since significant associations were observed between aortic stiffness and *ACAN* and *FBLN-1* gene variants in the primary and replication studies in the ENIGMA cohort, we investigated the actual role of these proteins in the vasculature by using ‘the aortae’, and performed SNP genotyping, gene and protein expression studies. Additionally, immunohistochemical / immunoflourescence studies were also carried to identify the tissue distribution in this arterial tissue (Fig. 1).

### Aortic tissue sample collection

Human aortic tissue samples (n = 232) collected from organ donors through our donor transplant coordinators at the Addenbrooke’s Hospital, Cambridge, were handled in accordance with the policies and procedures of the Human Tissue Act (UK) and with the approval of the Local and Regional Ethics Committees (MREC/03/2/074). Informed consent was taken from a family member, and all experiments performed in accordance with the relevant guidelines and regulations. Each arterial specimen was trimmed of blood vessels, any surrounding tissue and fat, and cut into 2 cm rings for biophysical measurements. From each specimen, some tissue was taken for DNA analysis, some preserved in RNA*later*® Stabilization Solution (Ambion^TM^ #AM7021) for RNA and some for protein extractions. A further sample was fixed in formalin and paraffin embedded (FFPE) for immunohistochemical investigations.

### Biophysical measurements and study design

Aortic stiffness was measured as Young’s Elastic Modulus (EM)^66^, *ex vivo* using the tensile test machine (Instron® model 5500 R, United Kingdom) in 220 donor aortic rings. Briefly, each ring was cycled 5 times in the range of 0–200 mmHg at a rate of mm*min-l. EM at a load of 100 mmHg was used to calculate PWV for each aorta via the Moens-Kortweg equation: $\sqrt{{\rm{EM}}\times \frac{{h}}{2{r}{\rm{\rho }}}}$, where h is the wall thickness, r is arterial radius measured with a digital callipers and ρ is blood density taken as 1.05 g/cm^3^. Study design for validation studies is illustrated in Fig. 1. For this *ex vivo study*, ascending and descending thoracic aortic samples from185 donors were used, as these segments differ histologically and embroyologically from the rest of the aorta. Of these donors, less than 10% were treated for CVD. We adopted the extreme-phenotype study design^67^, and pre-selected samples for lowest (n = 32) and highest (n = 28) EM values. Donors with EM readings of <0.1 MPa were grouped as ‘Low EM’ and those with EM readings of ≥0.25 MPa were grouped as ‘High EM’. Similarly, where donor aortae were stratified by age, individuals ≤32 years (n = 10) were classified as ‘Young’ and those >60 years (n = 10) were classified as ‘Old’.

### Statistical Analysis

Data were analyzed using IBM SPSS Statistics (version 23) and GraphPad Prism (version 7) software. One-way analysis of variance (ANOVA) with post-hoc testing compared the differences for continuous parameters and *x*^2^ testing compared differences for categorical variables. SNPs were tested for their association with aPWV by stepwise linear regression using an additive model after adjusting for confounding factors associated with aPWV, and the specific portion of variance explained by an R^2^ change. Unpaired t-test with Welch’s correction were used to compare differences between the two homozygous allele carriers of SNPs and their transcript levels. All the values are expressed as means ± SD in tables, and means ± SEM in figures. A probability value of <0.05 was considered significant.

For ACAN and FBLN-1 western blot quantifications, statistical significance between groups was determined using non-parametric Mann-Whitney U test. Fold difference in *ACAN* and *FBLN-1* gene expression between low and high EM donor samples was calculated using the 2^−ΔCt^ method where ΔCt = *ACAN* Ct – GAPDH Ct or *FBLN-1* Ct – GAPDH Ct, and statistical significance between low EM and high EM samples determined using the two-tailed Student’s *t*-test^68^. A post-hoc ANCOVA analysis was performed to account for confounding effect of age between the two groups.

## Electronic supplementary material

Supplementary Material

Supplementary Table 1

Supplementary Table 2
