# Supplementary material for: The matrix proteins aggrecan and fibulin-1 play a key role in determining aortic stiffness
Source: Sci Rep. 2018 Jun 4;8:8550. doi: 10.1038/s41598-018-25851-5 (PMC5986773; doi:10.1038/s41598-018-25851-5)
Supplement: Supplementary file 1 — Supplementary Material [file 41598_2018_25851_MOESM1_ESM.pdf]

SUPPLEMENTARY MATERIAL

The matrix proteins aggrecan and fibulin-1 play a key role in determining aortic stiffness

Yasmin, Raya Al Maskari, Carmel M. McEniery, Sarah E. Cleary, Ye Li, Keith Siew,

Nichola L. Figg, Ashraf W. Khir, John R. Cockcroft, Ian B. Wilkinson,

Kevin M. O'Shaughnessy

SUPPLEMENTARY METHODS

Genes, TagSNPs and Genotyping in ENIGMA. The set of genes and SNPs investigated in this study were purposefully selected from Durier et al.'s gene profiling work [1] and other published candidate gene association studies linked to arterial wall properties. [2-24] Attempts were made to include SNPs with prior evidence of putative function such as MMP-9 promoter polymorphism and/or those associated/implicated with arterial wall structure, function, inflammation and calcification. In this study, SNP selection encompassed a 2kb region on either side of the gene to include promoter, intronic and exonic variants from HapMap project and other public databases for European Ancestry population.

TagSNPs were generated using Paul de Bakker's Tagger [25] (pairwise tagging) and Gabriel et al. [26] algorithms on a block-by-block basis (linkage disequilibrium, LD) to capture genetic information for each gene using HapMap [27] and Haploview programs. [28] Where necessary, SNPs were also checked on the SeattleSNPs database. [29] We picked tag SNPs that covered larger regions and had r^2 cut-off of > 0.80.

Initially, 13461 tagSNPs identified from 53 genes were submitted to Illumina for checking and scoring. Based on the SNP Score, Designability Rank and Validation criteria, 384 tagSNPs were chosen for custom genotyping using GoldenGate assays. Since SNPs in close proximity along the DNA sequence can interfere with each other when assayed, care was taken to separate SNPs by more than 60bp on the Oligo Pool Assay. Ninety-five percent of the SNPs selected had SNP scores of >0.60 and Designability Rank of >1 which is deemed suitable for high assay success.

After genotyping and QC checks, we excluded 46 SNPs: 18 SNPs were not polymorphic, 4 had additional clusters (CNVs), 9 had no clusters or had one allele drop out, and 15 had low signal strength or bad cluster definition. A small number of subjects

with assay failures were also removed from subsequent analysis, hence the sample size differed for each SNP.

DNA and RNA extraction, quantification and quality assessment. Genomic DNA and total RNA was isolated from approximately 25mg of frozen aortic tissue samples. Tissues were mechanically disrupted using TissueLyser LT (QIAGEN #85600). DNA was extracted using the QIAamp DNA Mini Kit (QIAGEN #51304) following manufacturer's guidelines. Total RNA was extracted using Trizol Reagent (Ambion™ #15596-018) then purified with PureLink® RNA Mini Kit (Ambion™ #12183018) following manufacturer guidelines. DNA and RNA quantity and purity were determined using NanoDrop spectrophotometer (ND1000). A 260/280nm absorbance ratio of 1.7-1.9 for DNA and 1.9-2.1 for RNA were deemed suitable for downstream applications.

Genotyping in aortic sample. Two tagSNPs in *ACAN* (rs2882676, rs2293087) and two in *FBLN-1* (rs2018279, rs2238823) genes were genotyped using TaqMan® SNP Genotyping assays and TaqMan® Genotyping master mix (Thermo Scientific™ #4371355). Allelic discrimination was carried out by detecting allele specific fluorescence using the ABI 7500 Real Time PCR system as mentioned previously.

cDNA synthesis in aortic sample. First-strand cDNA was generated using the GoScript™ Reverse Transcription System (Promega #A5000). The reverse transcription reaction was performed on 1µg of total RNA primed with 0.5ug random primers and hybridized at 70°C for 5 min. The PCR thermal conditions consisted of an initial incubation at 22°C for 15 min, followed by 42°C for 60 min, 95°C for 5 min and finally 4°C for 5 min. cDNA was treated with RNase H (New England BioLabs #M0297) at 37°C for 20 min and samples were stored at -20°C until further analysis.

***ACAN* and *FBLN-1* gene expression analysis.** *ACAN* gene expression levels were quantified using TaqMan® probe and primers (assay ID: Hs00153936_m1). This assay overlaps exon boundaries 11-12, which code for keratin sulfate and chondroitin sulphate regions of human aggrecan core protein (NP_001126.3). *FBLN-1* gene expression was probed with TaqMan® probe and primers (assay ID Hs00972609_m1, Applied Biosystems™). Human GAPDH endogenous control (assay ID: 4326317E, Applied Biosystems™) was used to normalize the data. Real-time PCR was performed in duplicate using the TaqMan® Fast Advanced Master Mix (Thermo Scientific™ #4444557) in a total volume of 15ul using 1.5ul stock cDNA template.

Immunoblotting analysis. Frozen aortic tissue sections were homogenised using TissueLyser LT (Qiagen #85600) and protein lysates were extracted in NE-PER (Thermo Scientific™ #78833) lysis buffers containing protease inhibitors (Roche #11836170001). All steps were carried out at 4°C. Protein concentrations were determined with the Pierce™ BCA protein assay (Thermo Scientific™ #23225).

10ug of aortic protein lysates were incubated at 70^0C for 10min in Lodium dodecylsulfate (LDS) sample loading buffer (Novex™ #B0007) and Bolt® sample reducing agent (Novex™ #B0009). The protein was separated by SDS-gel electrophoresis in 4-12% gradient Bis-Tris Plus Bolt® gels (Life Technologies #BG04125BOX) at 200V for 30min and transfered to 0.22µM nitrocellulose membrane (Life Technologies #IB23001) using the iBlot2 dry blotting system (Novex™ #IB21001) at 20V for 7min. Prior to transfer, gels were equiliberated for 5min in NuPage transfer buffer (Novex™ #NP0006) containing 10% methanol. Membranes were blocked with 5% (wt/vol) milk in TBS buffer for 1h at room temperature then incubated with primary antibodies in TBS-Tween (0.1% Tween 20) containing 5% milk (wt/vol) for 16hr at 4^0C. Secondary antibodies were incubated in TBS-

Tween for 1h at room temperature in the dark. Membranes were then washed in TBS-T 3X for 15min each between primary and secondary antibody incubations and before visualization. Protein bands were detected using the Li-Cor Odessey System (Biosciences). Signal intensities were normalised against β-actin and quantified using ImageStudioLite software.

Immunohistological analysis. Immunohistochemical (IHC) staining was performed in formalin-fixed, paraffin-embedded (FFPE) aortic samples. Briefly, 4μm sections were deparaffinized in Histoclear (National Diagnostics #HS-200) then dehydrated through graduated alcohols. Antigen retrieval was performed by heating sections in R-Universal epitope recovery buffer (Aptum Biologics Ltd #AP0530-125) using 2100 Retriever (Aptum Biologics Ltd #R2100). EnVision™ + Dual Link system (Dako #K4063) was used for chromogenic detection of the primary antibodies. Sections were counterstained with haematoxylin (Sigma-Aldrich #MHS1), rehydrated through graduated alcohols, cleared in Histoclear then mounted with Histomount (National Diagnostics #HS-103).

Immunofluorescence analysis. FFPE sections were deparaffinised and antigen retrieval performed as described earlier. Sections were permeabilised with 0.05% v/v Triton X-100-PBS for 5 min and blocked for 2 h at room temperature with 5% v/v goat serum in 0.05% v/v Triton™ X-100–PBS. Sections were probed with the primary antibody for 16 h at 4°C in 2% v/v goat serum in 0.05% v/v Triton™ X-100–PBS. Slides were then washed for 5 min in 0.05% v/v Triton™ X-100–PBS and incubated in secondary antibody in 2% v/v goat serum in 0.05% v/v Triton™ X-100–PBS for 1 h at room temperature. Sections were counterstained with Sytox® Orange (ThermoFisher Scientific) at 1:10,000 in Milli-Q® water for 20 min at room temperature then mounted with ProLong® Gold Antifade Mountant (ThermoFisher Scientific).

Image acquisition and processing. Immunofluorescent images were acquired on the Leica TCS SP5 laser-scanning confocal with 488-, 561-, 633-nm laser lines mounted on an inverted Leica DMI 6000 CS fluorescent microscope using an HC PL FLUOTAR 20X/0.5NA objective with SP detectors set to 500-540nm (elastin autofluorescence), 590-610nm (sytox orange) and 645-738nm (Alexa 633) (Leica Microsystems Heidelberg GmbH, Germany). Acquisition parameters were as follows: 16-bit, 1024 × 1024 pixels, 400 Hz scan speed, 4-line Kalman filtering, sequential (by line) channel imaging, 1 airy unit pinhole, 13 slice z-stack with ~0.84 μm step size.

Immunohistochemically stained specimens were imaged in brightfield on an Olympus BX51 upright epifluorescent microscope using a UPLANFL 20×/0.5NA dry objective with the Infinity3 (Lumenera, Japan) CCD set to 1,936 × 1,456 pixels.

In FIJI image analysis software (http://fiji.sc/Fiji), fluorescent z-stacks underwent 8-bit conversion, background subtraction (100-pixel-radius rolling ball, no smoothing) and average intensity z-projection. Fluorescence and brightfield images were brightness and contrast adjusted using linear histogram stretching. Antibody signals across all samples were processed uniformly and in parallel to maintain comparability; while counterstain and autofluorescent signals were independently processed to enhance visibility. Images underwent bilinear rotational transformation and cropping to produce the final figures.

Antibodies used. Aggrecan G1 domain was probed with anti-aggrecan (Abcam #ab36861) rabbit polyclonal IgG antibody that recognizes residues 50-150 of aggrecan, used at 1:200 dilution. The chondroitin sulphate region was probed with aggrecan antibody (Bio-rad #MCA1451T) that recognizes an epitope within the N-terminal chondroitin sulphate-binding region at 1:400 dilution. Aggrecan G3 domain was probed with aggrecan antibody H-300 (Santa Cruz Biotechnology #sc25674) that recognizes residues 1911-1962

of aggrecan corresponding to the G3 domain at 1:100 dilution. Anti Fibulin-1 (Santa Cruz Biotechnology #sc-20818) rabbit polyclonal IgG antibody was used at 1:200 dilution. Beta-actin mouse monoclonal IgG (Thermo Scientific™ #MA5-15739) was used at 1:1000 dilution. Donkey anti-rabbit (Li-cor #925-32213) IRDye® 800CW and goat anti-mouse (Novex™ #A-21058) Alexa Fluor® 680 conjugated secondary antibodies were used for Western blots at 1:5000 dilution. Pre-absorbed goat IgG-conjugated Alexa Fluor® 633 secondary antibody (ThermoFisher Scientific, Waltham, MA, USA) was used for immunofluorescent staining at 1:200 dilution.

References

1 Durier, S. *et al.* Physiological genomics of human arteries: quantitative relationship between gene expression and arterial stiffness. *Circulation* **108**, 1845-1851, (2003).

2 Hanon, O. *et al.* Aging, carotid artery distensibility, and the Ser422Gly elastin gene polymorphism in humans. *Hypertension* **38**, 1185-1189 (2001).

3 Powell, J. T., Turner, R. J., Sian, M., Debasso, R. & Länne, T. Influence of fibrillin-1 genotype on the aortic stiffness in men. *J Appl Physiol (1985)* **99**, 1036-1040, (2005).

4 Liu, X., Wu, H., Byrne, M., Krane, S. & Jaenisch, R. Type III collagen is crucial for collagen I fibrillogenesis and for normal cardiovascular development. *Proc Natl Acad Sci U S A* **94**, 1852-1856 (1997).

5 Saito, Y. *et al.* Klotho protein protects against endothelial dysfunction. *Biochem Biophys Res Commun* **248**, 324-329 (1998).

6 Chen, J. Y. *et al.* Increased aortic stiffness and attenuated lysyl oxidase activity in obesity. *Arterioscler Thromb Vasc Biol* **33**, 839-846, (2013).

7 Akasaka, H. *et al.* A promoter polymorphism of lamin A/C gene is an independent genetic predisposition to arterial stiffness in a Japanese general population (the Tanno and Sobetsu study). *J Atheroscler Thromb* **16**, 404-409 (2009).

8 Yasmin *et al.* Matrix metalloproteinase-9 (MMP-9), MMP-2, and serum elastase activity are associated with systolic hypertension and arterial stiffness. *Arterioscler Thromb Vasc Biol* **25**, 372, (2005).

9 Medley, T. L., Kingwell, B. A., Gatzka, C. D., Pillay, P. & Cole, T. J. Matrix metalloproteinase-3 genotype contributes to age-related aortic stiffening through modulation of gene and protein expression. *Circ Res* **92**, 1254-1261, (2003).

10 Medley, T. L., Cole, T. J., Dart, A. M., Gatzka, C. D. & Kingwell, B. A. Matrix metalloproteinase-9 genotype influences large artery stiffness through effects on aortic gene and protein expression. *Arterioscler Thromb Vasc Biol* **24**, 1479-1484, (2004).

11 Yasmin *et al.* Variation in the human matrix metalloproteinase-9 gene is associated with arterial stiffness in healthy individuals. *Arterioscler.Thromb.Vasc.Biol.* **26**, 1799-1805 (2006).

12 Sie, M. P. *et al.* The interleukin-6-174 G/C promoter polymorphism and arterial stiffness; the Rotterdam Study. *Vasc Health Risk Manag* **4**, 863-869 (2008).

13 Trøseid, M. *et al.* Arterial stiffness is independently associated with interleukin-18 and components of the metabolic syndrome. *Atherosclerosis* **209**, 337-339, (2010).
14 Mahmud, A. & Feely, J. Adiponectin and arterial stiffness. *Am J Hypertens* **18**, 1543-1548, (2005).
15 Chen, W. *et al.* Nitric oxide synthase gene polymorphism (G894T) influences arterial stiffness in adults: The Bogalusa Heart Study. *Am J Hypertens* **17**, 553-559, (2004).
16 Yuan, M. *et al.* Genetic influences of beta-adrenoceptor polymorphisms on arterial functional changes and cardiac remodeling in hypertensive patients. *Hypertens Res* **29**, 875-881, (2006).
17 Palmieri, V. *et al.* Relation of fibrinogen to cardiovascular events is independent of preclinical cardiovascular disease: the Strong Heart Study. *Am Heart J* **145**, 467-474, (2003).
18 Bézie, Y. *et al.* [Molecular and cellular determinants of arterial stiffness: role of cell-matrix connections]. *Pathol Biol (Paris)* **47**, 669-676 (1999).
19 Yasmin *et al.* C-reactive protein is associated with arterial stiffness in apparently healthy individuals. *Arterioscler.Thromb.Vasc.Biol.* **24**, 969-974 (2004).
20 Naoum, J. J. *et al.* Lymphotoxin-alpha and cardiovascular disease: clinical association and pathogenic mechanisms. *Med Sci Monit* **12**, RA121-124 (2006).
21 Sie, M. P. *et al.* TGF-beta1 polymorphisms and arterial stiffness; the Rotterdam Study. *J Hum Hypertens* **21**, 431-437, (2007).
22 Kerr, P. G. & Guerin, A. P. Arterial calcification and stiffness in chronic kidney disease. *Clin Exp Pharmacol Physiol* **34**, 683-687, (2007).
23 Germain, D. P., Boutouyrie, P., Laloux, B. & Laurent, S. Arterial remodeling and stiffness in patients with pseudoxanthoma elasticum. *Arterioscler Thromb Vasc Biol* **23**, 836-841, (2003).
24 Mori, K. *et al.* Association of serum fetuin-A with carotid arterial stiffness. *Clin Endocrinol (Oxf)* **66**, 246-250, (2007).
25 de Bakker, P. I. *et al.* Efficiency and power in genetic association studies. *Nat Genet* **37**, 1217-1223, (2005).
26 Gabriel, S. B. *et al.* The structure of haplotype blocks in the human genome. *Science* **296**, 2225-2229, (2002).
27 Thorisson, G. A., Smith, A. V., Krishnan, L. & Stein, L. D. The International HapMap Project Web site. *Genome Res* **15**, 1592-1593, doi:10.1101/gr.4413105 (2005).
28 Smith, A. V. Manipulating HapMap Data Using HaploView. *CSH Protoc* **2008**, pdb.prot5025 (2008).
29 Carlson, C. S. *et al.* Selecting a maximally informative set of single-nucleotide polymorphisms for association analyses using linkage disequilibrium. *Am J Hum Genet* **74**, 106-120, (2004).

Supplementary Table 3. List of genes and SNPs associated with aPWV after bonferroni correction.

SNo	Gene Description	Locus Name	SNP IDs
1	Aggrecan 1 (chondroitin sulfate proteoglycan 1, large aggregating proteoglycan, antigen identified by monoclonal antibody A0122)	ACAN	rs2882676<br>rs2293087<br>rs3743399
2	Erythrocyte membrane protein band 4.1-like 2	EPB41L2	rs17059787
4	Fibulin-1	FBLN-1	rs2018279<br>rs2238823
4	Fibrinogen	FGB	rs2227401
5	Interleukin -18	IL18	rs795467
6	Integrin, alpha 6	ITGA6	rs13421350
7	Integrin, beta 3 (platelet glycoprotein IIIa, antigen CD61)	ITGB3	rs2292863
8	Matrix metalloproteinase-3	MMP-3	rs3025066
9	Nitric oxide synthase	NOS	rs3918169

Supplementary Table 4. Genotype differences in aPWV for all the 5 SNPs.

**SNPs**	**Genotypes**	**Study Populations**		
		Discovery	***Replication***	***Combined***
		Mean±SD	*Mean±SD*	*Mean±SD*
ACAN polymorphisms				
rs2882676	CC	6.10±1.23	5.66±0.67*	5.70±0.86
	CA	5.61±1.15	5.59±0.59	5.55±0.80
	AA	5.44±1.20	5.44±0.71	5.49±0.87
rs2293087	GG	6.10±1.24	5.64±0.65	5.69±0.84
	GT	5.63±1.19	5.56±0.67	5.52±0.83
	TT	5.41±1.17	5.55±0.61	5.45±0.79
rs3743399	GG	6.20±1.28	5.88±0.91*	5.94±1.01
	GA	5.73±1.25	5.61±0.63	5.65±0.85
	AA	5.40±1.19	5.56±0.62	5.56±0.82
FBLN-1 polymorphisms				
rs2018279	AA	5.89±1.25	5.67±0.68*	5.69±0.87
	AT	5.52±1.18	5.57±0.60	5.57±0.82
	AA	5.33±1.10	5.54±0.64	5.48±0.80
rs2238823	AA	6.35±1.25	5.62±0.65	5.69±0.86
	AG	5.80±1.18	5.59±0.65	5.62±0.82
	GG	5.30±1.10	5.43±0.58	5.34±0.72

* Homozygous alleles different at 0.05 significance level in the replication cohort.
